# Exploring the Effects of Volunteering on the Social, Mental, and Physical Health and Well-being of Volunteers: An Umbrella Review

**DOI:** 10.1007/s11266-023-00573-z

**Published:** 2023-05-04

**Authors:** Beth Nichol, Rob Wilson, Angela Rodrigues, Catherine Haighton

**Affiliations:** 1 Department of Social Work, Education, and Community Wellbeing, Northumbria University, Newcastle Upon Tyne, England; 2 Newcastle Business School, Northumbria University, Newcastle Upon Tyne, England; 3 Department of Psychology, Northumbria University, Newcastle Upon Tyne, England

**Keywords:** Umbrella review, Volunteering, Social prescribing, Wellbeing, Health

## Abstract

Volunteering provides unique benefits to organisations, recipients, and potentially the volunteers themselves. This umbrella review examined the benefits of volunteering and their potential moderators. Eleven databases were searched for systematic reviews on the social, mental, physical, or general health benefits of volunteering, published up to July 2022. AMSTAR 2 was used to assess quality and overlap of included primary studies was calculated. Twenty-eight reviews were included; participants were mainly older adults based in the USA. Although overlap between reviews was low, quality was generally poor. Benefits were found in all three domains, with reduced mortality and increased functioning exerting the largest effects. Older age, reflection, religious volunteering, and altruistic motivations increased benefits most consistently. Referral of social prescribing clients to volunteering is recommended. Limitations include the need to align results to research conducted after the COVID-19 pandemic. (PROSPERO registration number: CRD42022349703).

**Supplementary Information:**

The online version contains supplementary material available at 10.1007/s11266-023-00573-z.

## Introduction

Social prescribing is a person-centred approach involving referral to non-clinical services including those within the third sector (Public Health England, 2019), which describes groups or organisations operating independently to government, where social justice is the primary goal (Salamon & Sokolowski, 2016). It is an intervention that directs patients with non-medical health needs away from healthcare and towards social means of addressing their needs (Muhl et al., 2022), such as support with the social determinants of health including finance and housing, activities around art and creativity, and exercise (Thomson et al., 2015). Social prescribing can also involve referring clients to engage in volunteering (Thomson et al., 2015; Tierney et al., 2022), defined as unpaid work or activity to benefit others outside of the family or household, in which the individual freely chooses to participate (Salamon & Sokolowski, 2016). Volunteering, also known as community service in the USA, can be regular and sustained or ad hoc and short term (episodic) (Macduff, 2005) and encompasses activity directed towards helping others (civic) (Jenkinson et al., 2013), environmental conservation (environmental) (Husk et al., 2016), and as part of education (service learning), often accompanied by structured reflection of the voluntary activity (Conway et al., 2009).

Unique to other referrals within social prescribing, volunteering may provide a twofold benefit. Volunteering provides clear economic benefits to organisations (NCVO, 2021a) and acts as a ‘bridge’ of welfare services to deprived communities (South et al., 2011). There are also distinct benefits for recipients in comparison with professional help including increased sense of participation, self-esteem and self-efficacy, and reduced loneliness, due to a more neutral and reciprocal relationship (Grönlund & Falk, 2019). As utilised by social prescribing, volunteering as an intervention in itself is supported by clear health benefits to the volunteer, particularly improved mental health and reduced mortality (Jenkinson et al., 2013). There are many primary studies which find significant positive effects of volunteering on social, physical and mental health, including mortality and health behaviours (Casiday et al., 2008; Linning & Volunteering, 2018). Furthermore, there is evidence that these benefits occur from adolescence across the lifespan (Mateiu-Vescan et al., 2021; Piliavin, 2010), although they may increase with age (Piliavin, 2010). However, due to the poor quality of this evidence, it is unclear which of the benefits, particularly concerning mental health, predict rather than result from volunteering (Stuart et al., 2020; Thoits & Hewitt, 2001).

An investigation of the benefits of volunteering can therefore inform on the utility of this practice in improving the health and well-being of clients (Tierney et al., 2022) and support a twofold benefit (Mateiu-Vescan et al., 2021). Also, establishing the benefits may help retain volunteers within organisations (Mateiu-Vescan et al., 2021), as low volunteer retention (Chen et al., 2020) has been a key debated issue (Snyder & Omoto, 2008; Studer & Schnurbein 2023), with suggested solutions including maintaining motivation through opportunities for evaluation and self-development (Snyder & Omoto, 2008), improved management of volunteers (Studer & Schnurbein 2023), and recognising their value (Studer & Schnurbein 2023). However, outcomes of volunteering such as self-efficacy (Harp et al., 2017) and sense of connection (Dunn et al., 2021) have also been shown to predict retention.

An umbrella review methodology is appropriate to provide a systematic and comprehensive overview of the vast evidence on the benefits of volunteering and to determine which are most supported, making clear and accessible recommendations for research and policy (Pollock et al., 2020). An umbrella review can also help establish what works, where, and for whom, through comparison of different settings, volunteering roles, and populations from systematic reviews with different focuses (Smith et al., 2011). Thus, it is important that an exploration of the benefits of volunteering consider potential moderators. Umbrella reviews also assess the quality of the included systematic reviews and weight findings accordingly (Smith et al., 2011), which may help to establish a causal influence of volunteering. The emerging use of an umbrella review methodology in third sector research has enabled clear recommendations for practice, exploration of moderators and mediators, identification of gaps in the research, and recommendations for future reviews (Saeri et al., 2022; Woldie et al., 2018).

### Aims

The aims of this umbrella review were to;

1. Assess the effects of volunteering on the social, mental and physical health and well-being of volunteers, and;
2. Investigate the interactions between outcomes and other factors as moderators or mediators of any identified effects.

**Fig. 1:**
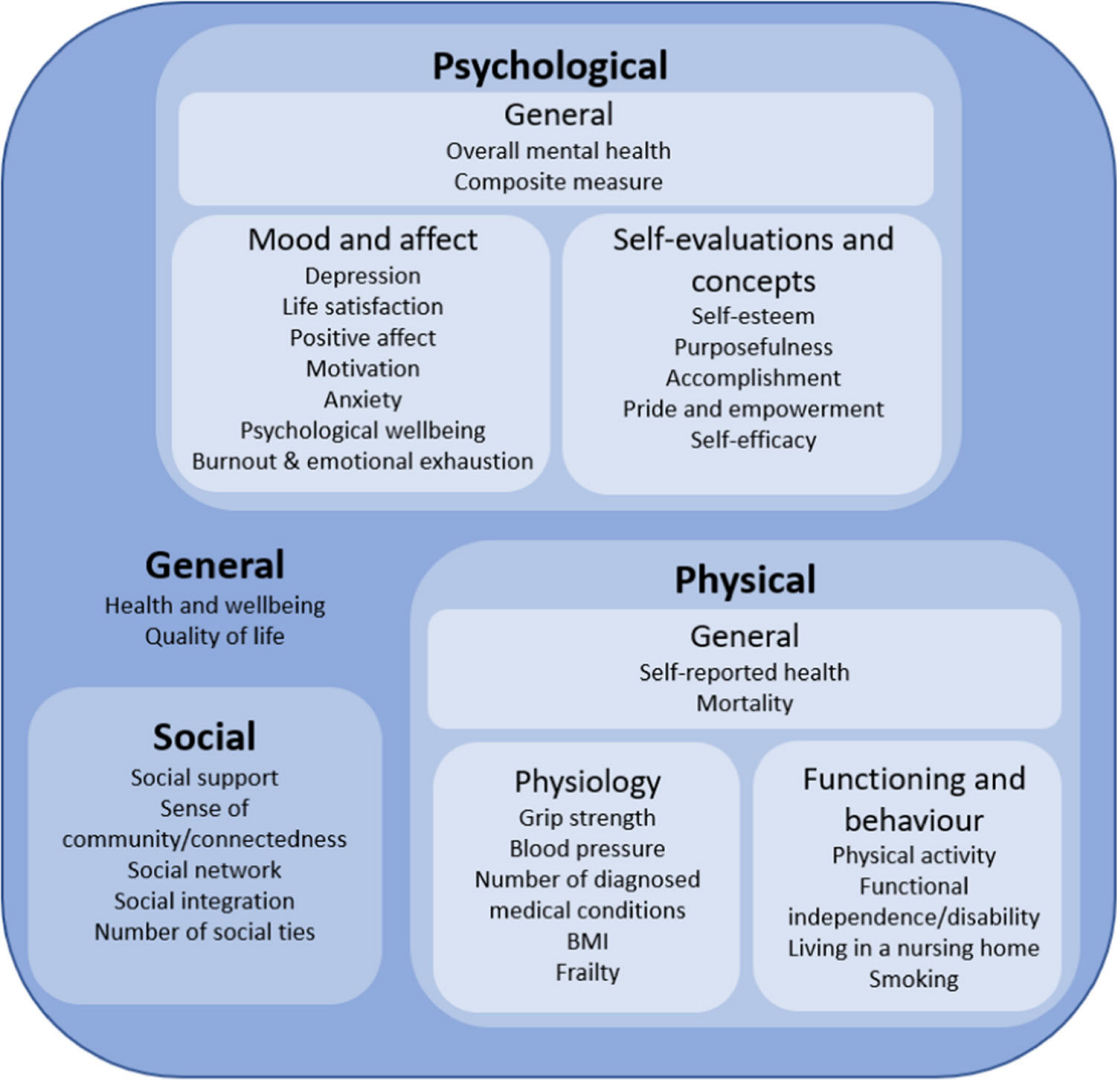
Outcomes identified and analysed within the current umbrella review, grouped by coding of outcome

Establishing clear conclusions to these aims helped identify gaps in the literature to direct future research and provided directions to support research and implementation of interventions involving volunteers. Specific outcomes explored within this review are displayed in Fig. 1.

## Methods

This umbrella review was pre-registered on the International Prospective Register of Systematic Reviews (PROSPERO) (Nichol et al., 2022) following scoping searches but prior to the formal research (registration number: CRD42022349703). Reporting of the umbrella review methodology followed the Preferred Reporting Items for Systematic Review and Meta-Analysis (PRISMA) (Page et al., 2020). Prior to formulating the research question, the International Prospective Register of Systematic Reviews (PROSPERO), the Joanna Briggs Institute (JBI) Systematic Review Register, and the Open Science Framework Registry were checked for pre-registrations of umbrella reviews of the same or a similar topic. No such umbrella review protocols were retrieved.

### Inclusion Criteria

#### Intervention: Volunteering

Volunteering was defined as conducting work or activity without payment, for those outside of the family or household. Participants of all ages were included. There were no limits by country or organisation or group that the volunteering was for. Although part of the definition of volunteering is that it is sustained (Salamon & Sokolowski, 2016), all durations of volunteering were included in this review to ensure a comprehensive search. Additionally, only reviews of volunteering involving some interpersonal contact with other volunteers or recipients were included. Reviews of volunteering in disaster settings such as warzones and aid for natural disasters were excluded, as these represent volunteering in extreme circumstances that is unusual and highly stressful (Thormar et al., 2010).

Systematic reviews were required to investigate the effect of volunteering on the volunteer. Reviews were excluded if volunteering was a component of a wider intervention. Reviews only assessing the effect of volunteering on the recipient were also excluded. The distinction between volunteer and recipient was sometimes less clear for reviews assessing the effect of intergenerational programmes. In this case, outcomes were only extracted for the group(s) that were performing work or activity, and no data was extracted from primary studies where neither group were.

#### Outcomes

The outcome of interest was health and well-being. This was categorised into general, psychological, physical, and social. Of additional interest was the interaction between these effects and with other factors such as demographics or factors associated with volunteering such as duration and type. Outcomes could be self-reported, or objective for physical outcomes (e.g. body mass index (BMI)). Reviews that did not assess effect were excluded, such as those exploring implementation, feasibility, or acceptability of volunteering as an intervention.

#### Types of Studies

The focus of this umbrella review was on systematic reviews of quantitative studies with or without meta-analyses to assess effect, although reviews of mostly quantitative studies were also included. The adopted definition of a systematic review was a documented systematic search of more than one academic database. Primary studies, reviews of qualitative or mostly qualitative literature, opinion pieces and commentaries were excluded.

### Search Strategy

The search was conducted on the 28th July 2022 via 11 databases including EPISTEMONIKOS, Cochrane Database, and PsychARTICLES, ASSIA and the Health Research Premium collection via ProQuest (Consumer Health Database, Health & Medical Collection, Healthcare Administration Database, MEDLINE®, Nursing & Allied Health Database, Psychology Database and Public Health Database). The search was applied to title and abstract and restricted to peer-reviewed systematic reviews published in English, as all reviewers were English language speakers with no translation services available. Initial scoping searches helped to build the search strategy (Supplementary Material 1). To maximise scope, forward and backward citation searching was applied, and the results of scoping searches and further sources such as colleagues and other academics were combined into the final umbrella review.

### Study Selection

Search results were exported via a RIS file and uploaded onto Rayyan for screening. Reviewer BN screened all reviews by title and abstract against the inclusion criteria, before screening the remaining (not previously excluded) articles based on full text. Details on independent screening and inter-rater reliability are available in Supplementary material 2.

### Quality Appraisal

Quality was assessed using the AMSTAR 2 checklist (Shea et al., 2021), which is designed to assess the quality of quantitative systematic reviews of healthcare interventions (Shea et al., 2021) and has the highest validity in comparison to other quality assessment tools (Gianfredi et al., 2022). Also, the accompanying guidance sheet ensures consistent use across reviewers. The 16 checklist items are presented under Table 1. Further details on quality appraisal for both the included reviews and primary included studies are available in Supplementary Material 3.

**Table 1:** Quality of the included reviews, as rated using the AMSTAR 2

Study	Q1	Q2	Q3	Q4	Q5	Q6	Q7	Q8	Q9	Q10	Q11	Q12	Q13	Q14	Q15	Q16
Anderson et al. (2014)	Y	N	Y	N	N	N	N	Y	N	N	N/A	N/A	N	Y	N/A	N
Blais et al. (2017)	Y	N	N	N	N	N	N	Y	N	N	N/A	N/A	N	N	N/A	N
Cattan et al. (2011)	Y	N	N	N	N	Y	N	N	N	N	N/A	N/A	N	Y	N/A	Y
Chen et al. (2020)	Y	N	N	PY	Y	Y	Y	Y	Y	N	N/A	N/A	N	Y	N/A	Y
Conway et al. (2009)	Y	N	N	N	N	Y	N	N	N	N	N	N	N	Y	N	N
Farrell & Bryant (2009)	N	N	N	PY	N	N	N	Y	N	N	N/A	N/A	N	Y	N/A	N
Filges et al. (2020)	Y	Y	Y	Y	Y	Y	Y	Y	Y	N	Y	Y	Y	Y	Y	Y
Galbraith et al. (2015)	Y	N	Y	N	Y	N	N	Y	N	N	N/A	N/A	N	N	N/A	N
Giraudeau & Bailly (2019	N	N	Y	Y	Y	N	N	Y	N	N	N/A	N/A	N	Y	N/A	N
Goethem et al., (2014)	Y	N	Y	Y	N	Y	N	N	N	N	Y	N	N	Y	Y	N
Gualano et al. (2018)	Y	N	Y	N	Y	Y	N	PY	Y	N	N/A	N/A	Y	Y	N/A	Y
Höing et al. (2016)	Y	N	N	PY	N	N	N	Y	N	N	N/A	N/A	N	Y	N/A	Y
Howard & Serviss (2022)	Y	N	N	PY	Y	N	N	N	N	N	N	N	N	N	Y	N
Hui et al. (2020)	Y	N	N	N	N	Y	N	N	N	N	Y	N	N	Y	Y	N
Hyde et al., (2014)	Y	N	N	N	Y	Y	N	Y	N	N	N/A	N/A	N	Y	N/A	Y
Jenkinson et al., 2013	Y	Y	Y	PY	Y	Y	N	Y	Y	N	N/A	N/A	Y	Y	N/A	Y
Kragt & Holtrop (2019)	N	N	Y	PY	N	N	N	N	N	N	N/A	N/A	N	Y	N/A	N
Lovell et al. (2015)	Y	Y	Y	N	Y	N	N	Y	N	N	N/A	N/A	N	N	N/A	Y
Manjunath & Manoj (2021)	Y	N	N	N	N	N	N	N	PY	N	N/A	N/A	Y	N	N/A	Y
Marco-Gardoqui et al. (2020)	Y	N	Y	PY	Y	Y	N	Y	N	N	N/A	N/A	N	Y	N/A	Y
Milbourn et al. (2018)	Y	N	N	N	N	N	N	PY	PY	N	N/A	N/A	Y	N	N/A	Y
O’Flynn et al. (2021)	Y	N	N	Y	N	N	N	N	N	N	N/A	N/A	N	Y	N/A	N
Okun et al. (2013)	Y	N	N	N	N	Y	N	PY	N	N	Y	N	N	Y	Y	N
Onyx & Warburton (2003)	N	N	N	N	N	N	N	N	N	N	N/A	N/A	N	Y	N/A	N
Owen et al., (2022)	Y	Y	N	N	Y	N	N	Y	PY	N	N/A	N/A	Y	Y	N/A	Y
Bonsdorff & Rantanen (2011)	Y	N	N	N	N	N	N	Y	N	N	N/A	N/A	N	Y	N/A	N
Wheeler et al (1998)	N	N	N	PY	N	N	N	Y	N	N	N	N	N	Y	Y	N
Willems et al. (2020)	Y	N	N	N	Y	Y	N	Y	PY	N	N/A	N/A	N	Y	N/A	Y

Q1: Did the research questions and inclusion criteria for the review include the components of PICO?

Q2: Did the report of the review contain an explicit statement that the review methods were established prior to the conduct of the review and did the report justify any significant deviations from the protocolreview?

Q4: Did the review authors use a comprehensive literature search strategy

Q5: Did the review authors perform study selection in duplicate?

Q6: Did the review authors perform data extraction in duplicate?

Q7: Did the review authors provide a list of excluded studies and justify the exclusions?

Q8: Did the review authors describe the included studies in adequate detail?

Q9: Did the review authors use a satisfactory technique for assessing the risk of bias in individual studies that were included in the review?

Q10: Did the review authors report on the sources of funding for the studies included in the review?

Q11: If meta-analysis was performed did the review authors use appropriate methods for statistical combination of results?

Q12: If meta-analysis was performed, did the review authors assess the potential impact of RoB in individual studies on the results of the meta-analysis or other evidence synthesis?

Q13: Did the review authors account for RoB in individual studies when interpreting/discussing the results of the review?

Q14: Did the review authors provide a satisfactory explanation for, and discussion of, any heterogeneity observed in the results of the review?

Q15: f they performed quantitative synthesis did the review authors carry out an adequate investigation of publication bias (small study bias) and discuss its likely impact on the results of the review?

Q16: Did the review authors report any potential sources of conflict of interest, including any funding they received for conducting the review?

### Data extraction and Synthesis

The data extraction form was created with guidance from Cochrane (Pollock et al., 2020). To increase transparency, data extraction was completed via SRDR plus, and made publicly available (https://srdrplus.ahrq.gov//projects/3274). Further information on data extraction, including on inter-rater agreement, is available in Supplementary Material 4.

### Data Analysis

The strategy of summarising rather than re-analysing the data of the reviews was adopted (Pollock et al., 2020). Vote counting by direction of effect was applied (McKenzie & Brennan, 2019), relying on the reporting of included systematic reviews. Variables were formed to allow for votes to be counted across reviews (e.g. self-esteem, self-efficacy and pride and empowerment were collapsed due to them regularly being combined by reviews). To test for significance, a two-tailed binomial test was applied with the null assumption that positive effects were of a 50% proportion (McKenzie & Brennan, 2019). Given that vote counting does not indicate magnitude of effect, results of meta-analyses are also presented. To estimate the degree of overlap of primary studies between the included reviews, the equation for calculated covered area (CCA) (Pieper et al., 2014) was applied. To prevent underestimating overlap, only primary studies addressing the effect of volunteering on the health of the volunteer were included when calculating overlap. Although vote counting also accounts for overlap, the resulting CCA was used as an additional tool for assessing the credibility of conclusions made.

## Results

### Search Outcomes

**Fig. 2:**
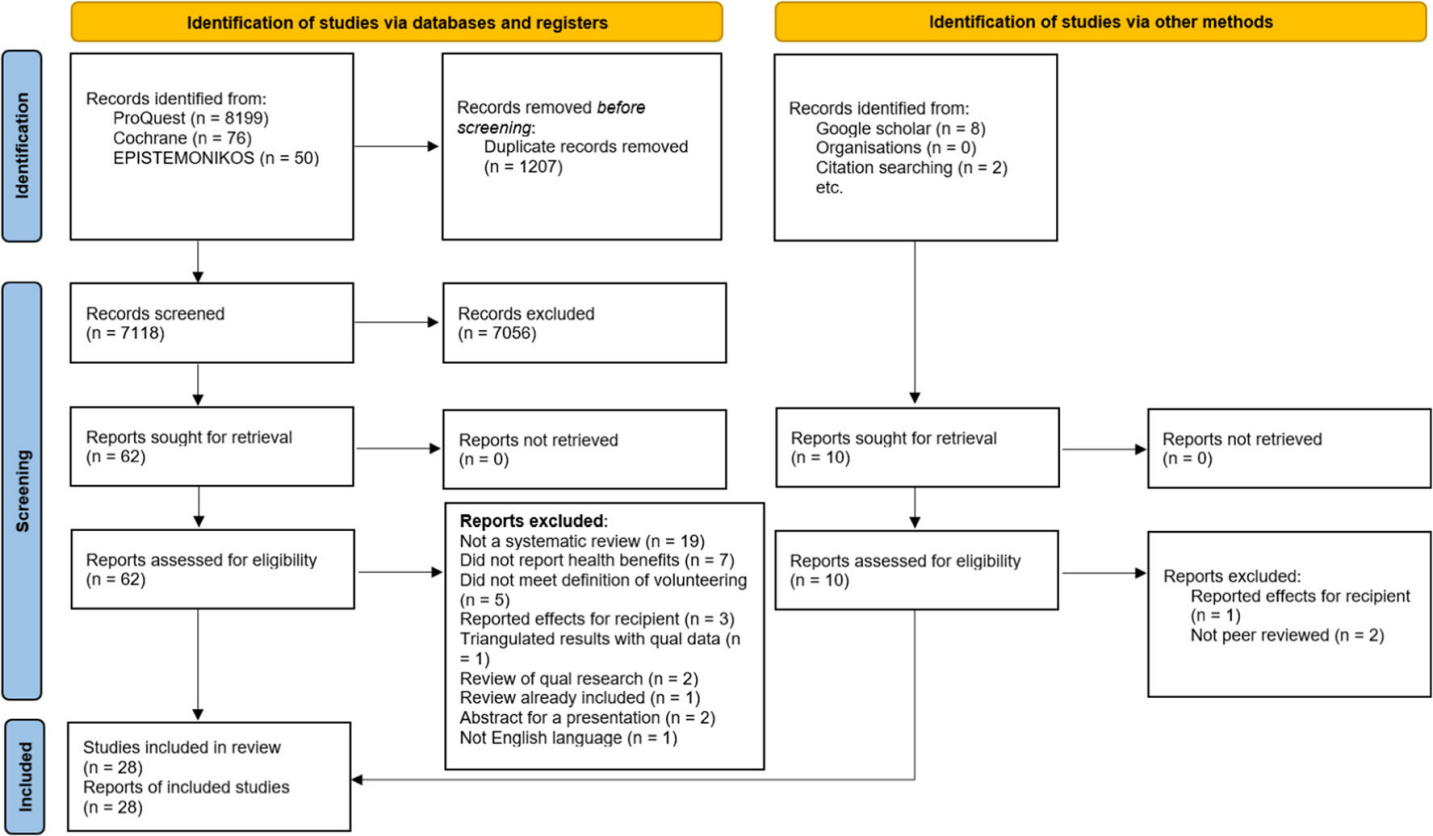
PRISMA flow diagram of retrieved articles (Page et al., 2020)

Initially 8325 articles were retrieved, as shown in Fig. 2. After removal of duplicates, 7118 remained for screening based on title and abstract and 62 articles remained to screen based on full texts, of which 21 reviews were included in the final review. A further 10 articles were retrieved from google scholar and citation searching, of which 7 were included, providing a total of 28 reviews. Excluded articles and the reasons for exclusion are available in Supplementary Material 5. Details on the inter-rater agreement of article screening can be found in Supplementary Material 6.

### Overlap

Authors of three included reviews were contacted to gain sufficient information to accurately calculate overlap, for example to separate studies of volunteering from those on prosociality in general (Goethem et al., (2014); Howard & Serviss, 2022; Hui et al., 2020). For one review (Goethem et al., (2014)), sufficient information to calculate true overlap was not obtained and thus it was excluded from the calculation of CCA. The excluded review was the only one that focused on adolescents; thus the exclusion is more likely to result in a conservative estimate of overlap rather than an underestimation. Despite this, CCA was 1.3%, indicating slight overlap. The overlap table used to calculate CCA is available from the corresponding author on request.

### Methodological Quality of Included Primary Studies

Only 12 of the included reviews assessed primary studies for quality or risk of bias (Chen et al., 2022; Filges et al., 2020; Gualano et al., 2018; Hui et al., 2020; Hyde et al., 2014; Jenkinson et al., 2013; Lovell et al., 2015; Manjunath & Manoj, 2021; Marco-Gardoqui et al., 2020; Milbourn et al., 2018; Owen et al., 2022; Willems et al., 2020). The tools most commonly used to assess study quality were the Effective Public Health Practice Project tool (Lovell et al., 2015; Owen et al., 2022) and JBI checklists (Manjunath & Manoj, 2021; Marco-Gardoqui et al., 2020). Those that assessed risk of bias mainly utilised Cochrane tools ROB-2 for randomised controlled trials (RCTs) (Gualano et al., 2018; Jenkinson et al., 2013), and ROBINS-I for non-RCTs (Chen et al., 2022; Filges et al., 2020; Gualano et al., 2018). Only two reviews removed studies from the narrative review (Milbourn et al., 2018) or meta-analysis (Filges et al., 2020) based on quality. Reported study quality varied, but most often was reported as mainly poor quality or high risk of bias.

### Methodological Quality of Included Reviews

As shown in Table 1, the quality of included reviews varied hugely. Only seven reviews scored more than 50% (Chen et al., 2022; Filges et al., 2020; Gualano et al., 2018; Jenkinson et al., 2013; Marco-Gardoqui et al., 2020; Owen et al., 2022; Willems et al., 2020). One review was found to be significantly higher quality than the rest (Filges et al., 2020). None of the included reviews reported the funding source of the included studies, and most did not report a pre-registration or protocol, or reference to excluded studies.

### Characteristics of Included Reviews

The main characteristics of included reviews are displayed in Table 2. Publication of reviews spanned from 1998 (Wheeler et al., 1998) to 2022 (Chen et al., 2022; Howard & Serviss, 2022; Owen et al., 2022), with search dates up to 2020 (Chen et al., 2022; Howard & Serviss, 2022; Owen et al., 2022). Most reviews focused on older people (Anderson et al., 2014; Bonsdorff & Rantanen 2011; Cattan et al., 2011; Chen et al., 2022; Filges et al., 2020; Gualano et al., 2018; Manjunath & Manoj, 2021; Milbourn et al., 2018; Okun et al., 2013; Onyx & Warburton, 2003; Owen et al., 2022; Wheeler et al., 1998), with inclusion criteria ranging from aged over 50 years (Anderson et al., 2014; Cattan et al., 2011; Manjunath & Manoj, 2021; Milbourn et al., 2018) to a sample with a mean age of 80 years or above (Owen et al., 2022). Only one review focused specifically on adolescents (Goethem et al., (2014)). The number of included primary studies included in the reviews ranged from 5 (Blais et al., 2017) to 152 (Kragt & Holtrop, 2019), although not all related to the benefits of volunteering. For those that reported on location of included samples, most reviews included participants mostly from the USA (Anderson et al., 2014; Blais et al., 2017; Bonsdorff & Rantanen 2011; Cattan et al., 2011; Farrell & Bryant, 2009; Filges et al., 2020; Giraudeau & Bailly, 2019; Gualano et al., 2018; Jenkinson et al., 2013; Marco-Gardoqui et al., 2020; Milbourn et al., 2018; Okun et al., 2013; Onyx & Warburton, 2003; Owen et al., 2022; Wheeler et al., 1998), followed by North America (Anderson et al., 2014; Blais et al., 2017; Hyde et al., 2014; Jenkinson et al., 2013), the UK (Farrell & Bryant, 2009; Lovell et al., 2015), and Australia (Kragt & Holtrop, 2019; Onyx & Warburton, 2003). Four reviews focused on intergenerational programmes (Blais et al., 2017; Galbraith et al., 2015; Giraudeau & Bailly, 2019; Gualano et al., 2018), two on service learning (Conway et al., 2009; Marco-Gardoqui et al., 2020), and five on specific roles including crisis line (Willems et al., 2020), environmental conservation (Chen et al., 2022; Lovell et al., 2015), care home work (Blais et al., 2017), and water sports inclusion (O’Flynn et al., 2021). One review limited the search to volunteering at a frequency less than seasonally (Hyde et al., 2014).

**Table 2:** Characteristics of included reviews

Review	Scope of the review	Search dates	Number of included studies	Population	Exclusion criteria for participants	Criteria for volunteering	Coding of outcomes assessed	Meta-analysis	AMSTAR 2 rating
Anderson et al. (2014)	The benefits of volunteering for older adults and build a theoretical model of how volunteering reduces risk of developing dementia	Inception up to April 2014	73	Mostly based in the USA and Canada, aged between 41 and 93	Older adults aged 50 or over	Formal volunteering	Psychological Physical Social General	No	− 10
Blais et al. (2017)	The benefits of intergenerational volunteering by students and residents of long-term care homes	Not provided	5	Based in the USA and Canada, mostly university students	High school or postsecondary volunteers, working with older adults residing in long-term care homes	Volunteering inside the long-term care homes and involved direct contact with the residents. Excluded service learning	Social	No	− 18
Cattan et al. (2011)	The impact of volunteering on older volunteers’ quality of life	Between 2005 and 2011	21	Mainly based in the USA and included participants from either the age of 55 or 65 years	Older adults aged 50 years or over	Formal volunteering	Psychological Physical General	No	− 10
Chen et al. (2020)	The benefits, motivations and drawbacks of environmental volunteering in older adults	Inception to July 2020	9	SS of 328, most based in Taiwan or the USA. Mean age ranged from 65.6 to 75.7	Older adults	Volunteering with an intention to improve the outdoor environment	Psychological Physical Social General	No	9
Conway et al. (2009)	Changes associated with service learning and moderators of these changes	Inception to June 2008	103	SS of 1,819 for self-evaluations, and 274 for well-being	None	Service learning	Psychological General	Yes	− 20
Farrell & Bryant (2009)	Volunteering to promote social inclusion for volunteers with mental health problems	Not provided	14	Mainly based in the UK or USA, range of subpopulations (e.g. people with disabilities)	Participants with mental health problems	Volunteering to promote social inclusion	Psychological Social General	No	− 15
Filges et al. (2020)	The effects of volunteering on physical and mental health adults aged over 65	Inception to December 2018, more searches carried out in September and October 2019	90 (26 for this data synthesis)	Average SS of 2,369 for volunteers, and an average of 61% female. Mostly from the USA, average age of 76 for volunteers	Older adults aged 65 or over	Formal volunteering in comparison to non-volunteers	Psychological Physical General	Yes	30
Galbraith et al. (2015)	The goals, characteristics, and outcomes of intergenerational programmes for children or youth and people with dementia	Inception to February 2014	27	No information (only studies were of volunteering)	People with dementia and participants aged under 19	Dementia specific intergenerational programmes	Psychological	No	− 10
Giraudeau & Bailly, 2019	Characteristics, definition, and benefits of intergenerational programmes for school-aged children and adults aged above 60 years	2005–2015	11	SS ranged from 11 to 46 for older volunteers, mostly based in the US	Older adults aged 60 or over and school-aged children	Intergenerational programmes	General	No	− 6
Goethem et al., (2014)	The general, academic, personal, social, and civic outcomes of community service, and their moderators including reflection	1980 and September 2012	49	No information	Adolescents between 12 and 20 years old without a mental disability	Volunteering, community service, and service learning	Psychological Social	Yes	− 4
Gualano et al. (2018)	The effects of intergenerational programmes on elders and children, and the key elements that determine their success	Not provided	27	SS of older adults ranged from 6 to 162, based mostly in the USA followed by Japan	Older adults and school or pre-school children	Intergenerational programmes	Psychological Physical Social General	No	9
Höing et al. (2016)	To support the development of policy and selection of volunteers working with medium to high risk sex offenders	1999 to October 2012	50	Most either focused on adults in general, or older adults aged 55 or over	For volunteering with sex offenders: working with sec offenders with the aim of reducing the behaviour	Volunteering in general and volunteering for medium to high risk sex offenders	Psychological Physical Social General	No	− 7
Howard & Serviss (2022)	Benefits of corporate volunteering programmes, and whether individual or organisational-level participation is most beneficial	Inception to May 2020	57	No information	Individual or organisational level	Corporate volunteering programmes	Psychological General	Yes	− 1
Hui et al., (2020)	Strength of the prosociality to well-being link under different conceptualisations, and their moderators	Inception to April 2014, more searches conducted in December 2016 and September 2019	126	No information	Adults 18 or over	Prosociality variables (including volunteering)	General	Yes	− 12
Hyde et al., (2014)	Benefits of episodic volunteering	Inception to April 2014	41 overall (20 within health and social welfare)	Mostly based in North America, most common age range was 30–60, mostly Caucasian, married, employed, and of middle income	None	Episodic volunteering outside of disaster settings and within one’s country (once or on a seasonal or annual basis)	Social	No	− 2
Jenkinson et al., 2013	Benefits of formal volunteering for physical and mental health and survival, and the influence of volunteering type and intensity	Inception to January 2013	40	Mostly based in the USA and North America and recruited those 50 years or over. Total SS of 308 for RCTs and 307 for NRCTs, and most cohort studies recruited samples over 1000	Adults aged 16 or over	Formal volunteering (sustained and regular: over 1 h twice monthly)	Psychological Physical General	No	17
Kragt & Holtrop (2019)	Characteristics, motivations, benefits, psychological contract, commitment, and withdrawal of volunteering in Australia	Inception to August 2018	152 (it total, on all aspects of volunteering)	All based in Australia	Participants in Australia	None	Psychological Social General	No	− 15
Lovell et al. (2015)	Impact of participation in environmental enhancement and conservation activities on health and well-being	Inception to October 2012	23 (13 with quantitative data)	Mostly based in the UK with samples aged between 40 and 60. SS ranged from 3 to 2630	None	Volunteering: outdoor and physically active environmental enhancement or conservation	Psychological Physical Social General	No	0
Manjunath & Manoj (2021)	Effectiveness of interventions to decrease social isolation in older adults	No information	20	2 studies eligible for volunteering; 1 international, the other based in Sweden	Adults aged 50 or over	Interventions to reduce isolation targeted towards older adults experiencing loneliness (included volunteering)	Psychological	No	− 11
Marco-Gardoqui et al. (2020)	The academic, personal, and social impact of service learning on students in business schools	Inception to October 2019	32	Mean SS of quant studies was 228. Mostly based in the USA. No first year students	Business students	Service learning	Psychological Social	No	5
Milbourn et al. (2018)	The relationship between time spent volunteering and quality of life in adults aged over 50	January 2000 to April 2014	8	SS ranged from 180 to 4860, mostly based in the USA, women, Caucasian, with a variety of income and education levels	Adults aged 50 or over	Time spent volunteering	Psychological Physical Social	No	− 8
O’Flynn et al. (2021)	The motivation and benefits of volunteers in inclusive watersports	Not provided	8 for benefits	No information	None	Volunteers in sport or disability inclusion	Social General	No	− 14
Okun et al. (2013)	The relationship between organisational volunteering and mortality in adults aged over 55	Inception to November 2011	13	Mainly based in the USA. SS ranged from 868 to 15,938. Median age was 66.5 years	Older adults	Organisational volunteering	General	Yes	− 9
Onyx & Warburton (2003)	To investigate the relationship between volunteering and health among older people	Not provided (searched last 10 years)	25	Developed countries, mostly the USA and Australia	Older adults	Volunteering	Psychological Physical General	No	− 22
Owen et al., (2022)	The effectiveness of purposeful activity on well-being and quality of life outcomes in ‘oldest old’ adults (aged over 80)	Inception to April 2020	8 (5 for volunteering)	Mostly from the USA, SS ranged from 10 to 88	Older adults with a mean age of 80 or above	Purposeful activity (divided into volunteering and learning a new skill)	Psychological General	No	5
Bonsdorff & Rantanen (2011)	The relationship between formal volunteering and well-being for older volunteers and the people they serve	Inception to November 2009	16	All based in the USA. SS ranged from 705 to 7496 for prospective studies, the SS for the included RCT was 128. Ages ranged between 60 and 97 for the prospective studies, mostly women and White, and were more highly educated and were of better perceived health than non-volunteers	Adults aged 60 or over	Volunteering in visits or within a timeframe	Psychological Physical General	No	-14
Wheeler et al (1998)	The effectiveness of volunteering for older adults and the people they serve	No information	37 (30 for outcomes of volunteers)	SS ranged from 15 to 2164 (median 98), mostly based in the USA. Average age was 71, mostly White (90%) and female (72%)	Older adults	All forms of volunteering	Psychological	Yes	− 17
Willems et al. (2020)	The mental well-being of crisis line volunteers and moderators	Inception to November 2018	13	SS ranged from 28 to 216 for the quantitative surveys. Sample were a range of ages and mostly female	Crisis line volunteers	Volunteers from a crisis line or chat line	Psychological Social	No	1

Several of the included meta-analyses, whilst employing a systematic search, did not perform any form of narrative synthesis alongside the results of the meta-analyses, meaning information about the characteristics of included studies was missing.

### Publication Bias

Seven of the included reviews applied a meta-analysis (Conway et al., 2009; Filges et al., 2020; Goethem et al., (2014); Howard & Serviss, 2022; Hui et al., 2020; Okun et al., 2013; Wheeler et al., 1998). Of these, five reported testing for publication bias (Filges et al., 2020; Goethem et al., (2014); Howard & Serviss, 2022; Hui et al., 2020; Okun et al., 2013; Wheeler et al., 1998). Generally, there was no strong evidence to indicate publication bias, although one review found a likelihood of publication bias specifically for the analyses of moderators on the risk of mortality (Okun et al., 2013). Also, one review reported three approaches to assess publication bias which gave mixed findings (Hui et al., 2020), and as the remaining reviews assessed publication bias in a variety of ways such as funnel plots (Filges et al., 2020), publication as a moderator (Goethem et al., (2014)), trim and fill procedure (Okun et al., 2013), and Rosenthal’s failsafe (Wheeler et al., 1998), results may not be reliable.

### Findings

Results of vote counting by direction of effect from the 18 included reviews are shown in Table 3. Five meta-analysis did not provide sufficient information to be included (Conway et al., 2009; Goethem et al., (2014); Howard & Serviss, 2022; Hui et al., 2020; Wheeler et al., 1998), and one only provided sufficient information to include one variable (Cattan et al., 2011).

**Table 3:** Summary table of direction and strength of evidence for each outcome, and strength of potential moderators and mediators

Coding of outcome	Outcome	Vote counting	Strength of evidence (vote counting, magnitude of effect indicated by included meta-analyses, overall judgement)	Moderators (amplifying effect) and mediators
General	Health outcomes overall	N/A	N/A	**Moderators** Structured reflection (use of) Age (older) SES (lower) Motivation (altruistic/intrinsic, religious) Social benefits (social connection, support and interaction) Optimal frequency (uncertain) Formality (uncertain)
	Well-being (general)	3 reviews with 7 unique studies were identified. All studies supported a positive effect, which was statistically significant (7/7; 100%, *p* = .016)	Moderate (consistent, magnitude of effect is small to very small)	**Moderators** Formality (informal/mixed volunteering) Motivations (prosocial) Recipient response (feeling appreciated) Level of participation (organisational level) Frequency (mostly consistent) **Mediators** Religiosity (partial)
	Quality of life	7 reviews with 15 unique studies were identified. A statistically significant majority of studies supported a positive effect (13/15; 87%, *p* = .007)	Moderate (consistent, meta-analysis required to determine magnitude)	**Moderators** Recipient response (feeling appreciated) **Mediators** Feeling appreciated
Psychological	Burnout and emotional exhaustion	3 reviews with 12 unique studies were identified. A statistically significant majority of studies supported a positive effect (11/12; 92%, *p* = .006)	Moderate (consistent specifically within emotionally demanding roles, meta-analysis required to determine magnitude of effect)	**Moderators** Age (younger) Role (emotionally demanding) Positive coping strategies (lack of) Social support (lack of) Education (lower) Empathy with recipient (empathising)
	Purposefulness and meaningfulness	6 reviews with 9 unique studies were identified. All studies supported a positive effect, which was statistically significant (9/9; 100%, *p* = .004)	Moderate (consistent, meta-analysis required to determine magnitude)	
	Life satisfaction	11 reviews with 30 unique studies were identified. A statistically significant majority of studies supported a positive effect (27/30; 90%, *p* < .001)	Strong (highly consistent, magnitude of effect is small)	**Moderators** Formality (formal volunteering) Recipient response (feeling appreciated) **Mediators** Social benefits
	Depression	11 reviews with 41 unique studies were identified. A statistically significant majority of studies supported a positive effect (39/41; 95%, *p* < .001)	Strong (highly consistent, magnitude of effect is very small)	**Moderators** Recipient response (feeling appreciated) Gender (women) Age (older) Empathetic arousal (low)
	Psychological well-being	10 reviews with 29 unique studies were identified. All studies supported a positive effect, which was statistically significant (29/29; 100%, *p* < .001)	Strong (highly consistent, meta-analysis required to determine magnitude)	
	Self-efficacy, self-esteem, and pride and empowerment	12 reviews with 43 unique studies were identified. A statistically significant majority of studies supported a positive effect (40/43; 93%, *p* < .001)	Strong (highly consistent, meta-analysis required to determine magnitude)	**Moderators (pride and empowerment)** SES (lower)
	Positive affect	7 reviews with 18 unique studies were identified. A statistically significant majority of studies supported a positive effect (16/18; 89%, *p* = .001)	Moderate (consistent, meta-analysis required to determine magnitude)	
	Motivation	2 reviews with 5 unique studies were identified. All studies supported a positive effect, although non-significant (5/5; 100%, *p* = .063)	Weak (insufficient evidence, meta-analysis required to determine magnitude)	
	Anxiety	3 reviews with 3 unique studies were identified. All studies supported a positive effect, although non-significant (3/3; 100%, *p* = .250)	Weak (insufficient evidence, meta-analysis required to determine magnitude of effect)	
	Mental health (general)	2 reviews with 5 unique studies were identified. Findings were inconsistent (3/5; 60%, *p* = 1.00)	Very weak (inconsistent/mixed, meta-analysis required to determine magnitude)	
Physical	Mortality	8 reviews with 30 unique studies were identified. All studies supported a positive effect, which was statistically significant (30/30; 100%, *p* < .001)	Very strong (highly consistent, effect was the second largest outcome in magnitude of the meta-analyses included)	**Moderators** Covariates (SES, age, religious attendance, social support and health habits)
	Maintenance of functional independence and reduced functional disability	7 reviews with 22 unique studies were identified. All studies supported a positive effect, which was statistically significant (22/22; 100%, *p* < .001)	Very strong (highly consistent, effects were the largest outcome in magnitude of the meta-analyses included)	
	Physical activity	7 reviews with 16 unique studies were identified. All studies supported a positive effect, which was statistically significant (16/16; 100%, *p* < .001)	Strong (highly consistent, meta-analysis required to determine magnitude of effect)	
	Self-reported health	10 reviews with 21 unique studies were identified. A statistically significant majority of studies supported a positive effect (18/21; 86%, *p* = .001)	Moderate (consistent, magnitude of effect is very small)	**Moderators** Type (environmental compared to civic) Frequency (mostly consistent)
	Grip strength	3 reviews with 3 unique studies were identified. All studies supported a positive effect, although non-significant (3/3; 100%, *p* = .250)	Weak (insufficient evidence, meta-analysis required to determine magnitude of effect)	
	Decreased smoking	1 review with 4 unique studies were identified. All studies supported a positive effect, although non-significant (4/4; 100%, *p* = .125)	Weak (insufficient evidence, meta-analysis required to determine magnitude of effect)	
	Blood pressure	1 review reported one study (1/1; 100%)	Weak (insufficient evidence, requires more research)	
	BMI		Weak (insufficient evidence, requires more research)	
	Frailty		Weak (insufficient evidence, requires more research)	
	Living in a nursing home	1 review reported one study (1/1; 100%)	Weak (insufficient evidence, requires more research)	
	Number of medical conditions	1 review reported one study (1/1; 100%)	Weak (insufficient evidence, requires more research)	
Social	Social network/ support	5 reviews with 12 unique studies were identified. A statistically significant majority of studies supported a positive effect (11/12; 92%, *p* = .006)	Moderate (consistent, meta-analysis required to determine magnitude of effect)	
	Social connectedness/ sense of community	5 reviews with 18 unique studies were identified. A statistically significant majority of studies supported a positive effect (17/18; 94%, *p* < .001)	Strong (highly consistent, meta-analysis required to determine magnitude of effect)	
	Social integration	2 reviews with 7 unique studies were identified. A majority of studies supported a positive effect, although non-significant (6/7; 86%, *p* = .125)	Weak (insufficient evidence, meta-analysis required to determine magnitude of effect)	
	General social benefits	1 review with 2 unique studies were identified. All studies supported a positive effect, although non-significant (2/2; 100%, *p* = .500)	Weak (insufficient evidence, requires more research)	
	Social ties	4 review with 4 unique studies were identified. All studies supported a positive effect, although non-significant (4/4; 100%, *p* = .125)	Weak (insufficient evidence, meta-analysis required to determine magnitude of effect)	

**Coding used to describe strength of the evidence:** Highly consistent: vote counting significant at the *p* < .001 level. Consistent; vote counting significant at the *p* = .05 level. Insufficient evidence; all in favour, but binomial test non-significant, Inconsistent: highly mixed. Magnitude of effect; small (OR of between .30 and .20), very small (OR below .10). Overall judgement: very strong (highly consistent, largest effect size), strong (highly consistent, small effect size), moderate (consistent, no pooled effect size determined or small to very small effect), weak (insufficient evidence), very weak (inconsistent evidence)

#### General Effects on Health and Well-being

Fifteen of the included reviews reported general effects on health and well-being (Table 4). Reviews reporting on composite, general measures of health mainly assessed well-being, although others measured quality of life. Generally, most reviews reported that volunteering improved well-being (Anderson et al., 2014; Cattan et al., 2011; Gualano et al., 2018; Hui et al., 2020; Jenkinson et al., 2013; Kragt & Holtrop, 2019; O’Flynn et al., 2021; Onyx & Warburton, 2003; Owen et al., 2022) and quality of life (Anderson et al., 2014; Cattan et al., 2011; Höing et al., 2016). However, the relationship with well-being was often small and with exceptions (Conway et al., 2009), and one review found most studies reported no significant impact on well-being or quality of life (Lovell et al., 2015), possibly because the review assessed environmental volunteering specifically. The review that reported on quality of life with the highest quality reported only significant positive relationships between volunteering and well-being and quality of life (Jenkinson et al., 2013), although there was evidence to suggest an impact on quality of life only when volunteers felt their contribution was appreciated (Jenkinson et al., 2013). One review found only organisational level and not individual level participation in volunteering to significantly increase well-being (Howard & Serviss, 2022), another found increased well-being for older but not younger people (Farrell & Bryant, 2009), and another found a curvilinear relationship such that a moderate intensity of volunteering was most beneficial (Bonsdorff & Rantanen 2011).

**Table 4:** General benefits

Review	Positive outcomes (number of studies)	Negative or non-significant outcomes	AMSTAR 2 rating
Anderson et al. (2014)	Increased well-being (2 prospective) Increased quality of life (2 descriptive, 2 cross-sectional, 1 prospective)		− 10
Cattan et al. (2011)	Increase in quality of life (CASP score) for older adults (4) Increased self-rated health/mental health (8) Increased physical/mental health (7)		− 10
Conway et al. (2009)		Negligible effect on well-being with a CI that crossed 0	− 20
Farrell & Bryant (2009)	Protective effect against well-being in over 65 s (1)	No effect on well-being in younger age groups (1)	− 15
Gualano et al. (2018)	Significant increase in well-being (2)		9
Höing et al. (2016)	Increased quality of life (6)	No significant improvements in well-being (1)	− 7
Howard & Serviss (2022)	Significant prediction of organisational-level participation and well-being	No significant prediction of employee-level voluntary participation and well-being	− 17
Hui et al., (2020)	Small but significant prediction of both binary and continuous measures of volunteering with well-being Very small but significant prediction of formal volunteering and well-being		− 12
Jenkinson et al. 2013	Significantly increased well-being (1 RCT, 3 cohorts follow-ups between 10 and29 years) Improved quality of life when volunteers felt appreciated (2 cohorts)		17
Kragt & Holtrop (2019)	Improved well-being compared to non-volunteers (3) (a dose response relationship for older adults (2))		− 15
Lovell et al. (2015)	Increased quality of life (4)	Mostly non-significant effects on well-being, with small sample sizes, or inconsistent evidence Mixed evidence increased quality of life, 1 found a negative effect	0
O’Flynn et al. (2021)	Increased well-being (2)		− 14
Onyx & Warburton (2003)	Increased personal well-being (6) (several studies indicate a curvilinear relationship)		
Owen et al., (2022)	Increase in at least one well-being outcome (4)	No significant effect on well-being (1) Significant improvement in well-being also in the usual care group (1)	5
Bonsdorff & Rantanen (2011)	Curvilinear relationship with well-being; moderate is best (2)		− 14

#### Psychological Effects on Health and Well-being

Psychological effects were the most commonly reported health and well-being outcome of volunteering, reported by 23 reviews (Table 5). The reviews that reported on general mental health reported mixed findings (Farrell & Bryant, 2009; Lovell et al., 2015; Milbourn et al., 2018), likely due to the large variation in how mental health was defined and measured. Whilst some considered mental health to be a distinct factor (Farrell & Bryant, 2009; Lovell et al., 2015), others combined factors such as life satisfaction into a composite measure of mental health (Milbourn et al., 2018).

**Table 5:** Psychological benefits. Displayed in brackets are the number of primary included studies to support the review findings. Where no brackets are provided, findings are the result of meta-analyses

Review	Positive outcomes (number of studies)	Negative or non-significant outcomes	AMSTAR 2 rating
Anderson et al. (2014)	Improvement in mood in women but not men (1) Reduced depression (cross-sectional: 4, prospective cohort: 15) Increased positive affect or happiness (descriptive: 1, prospective: 4, cross-sectional: 5) Greater life satisfaction (descriptive: 2, cross sectional: 6, prospective cohort: 2) Improvements in self-esteem or a sense of mastery (descriptive: 5, prospective cohort 2) Feeling useful and self-fulfilled (1 descriptive) Greater resilience (1 cross sectional)	No association with happiness (1 cross-sectional, 1 prospective) No association with life satisfaction (1 cross-sectional although the timescale of volunteering was short, 1 prospective although the follow-up was long) No association with improvements in self-esteem or a sense of mastery (2 cross sectional studies, 1 prospective cohort)	− 10
Cattan et al. (2011)	Reduction in depression (6), in women but not men (1) Improved psychological well-being (1) Improved psychological well-being (3) Greater life satisfaction (2)		− 10
Chen et al. (2020)	Increased positive outlook/affect (2) Increased life satisfaction (1) Decreased distress (1) and depression (2) Increased happiness and optimism (1) Increased self-esteem (2) Increased purposefulness/usefulness (2) Increased motivation (1) For volunteering in recycling specifically: Increased self-compassion (2) Reduced depression (2) Increased happiness (2) Increased positive affect and decreased negative affect (1) Increased life satisfaction (1)		9
Conway et al. (2009)	Self-evaluations		− 20
Farrell & Bryant (2009)	Decreased depression in older adults (1) Increased mental health and well-being (1) Increased life satisfaction for adults with disabilities (1) improved confidence and feeling valued (1) Empowerment and pride for adolescents with disabilities (1) Built confidence (2) Increased satisfaction (1) Increased self-esteem (4) Increased empowerment in people with mental health problems (1)	No effect on depression in younger age groups (1) 22% reported a negative impact on their mental health (1)	− 15
Filges et al. (2020)	Small but significant overall decrease in severity of depression		30
Galbraith et al. (2015)	Children felt helpful (1) Older people: Increased sense of purpose and usefulness (2) Joy derived from teaching children (1) Increased confidence and self-esteem, feeling loved (1) Renewed sense of usefulness (2) Decreased anxiety (2) Increased positive affect (1)		− 10
Giraudeau & Bailly (2019)	Older adults: Increased empowerment score (1) Fewer depressive symptoms and better mental health (1)		
Goethem et al. (2014)	Small but significant effect on attitudes towards the self and personal competence Personal and self(related): concept, attitudes, preferences, experiences, motivations, well-being, self-efficacy (15)		− 4
Gualano et al. (2018)	Significantly increased meaningfulness (1) Significantly decrease in stress (1)	No significant changes in depressive symptoms (1)	9
Höing et al. (2016)	Volunteering in general: Increased self-reported happiness (2) Increased life satisfaction and less negative affect and depression (7) An improved sense of purpose and accomplishment (2) Increased empowerment and self-esteem (5) Volunteers for sex offenders: Witnessing the core member changing for the better increased satisfied feeling of reward (1)	Decreased life satisfaction (1), overburdening and strain with high hours volunteering (1) Emotional exhaustion and burnout symptoms (6) (although these were generally not alarming symptoms (3)) Volunteering with sex offenders: Stress, rumination, worries of risk and feeling unsafe (1) Volunteers for sex offenders: Doubts about the motivation and effort of the core member produced emotional stress, irritation, frustration, and hopelessness (1) Increased depression and emotional problems when volunteering involved empathic over-arousal (e.g., in HIV— caregiving) (1)	− 7
Howard & Serviss (2022)	Significantly increased job satisfaction with organisational-level volunteering participation	No significant increase in life satisfaction with employee-level volunteering participation	− 17
Jenkinson et al., 2013	Significantly increased empowerment (1) Significantly decreased stress (1) Decreased levels of depression (4 cohort) Improved life satisfaction (4 cohorts) (follow-ups between 3 and 25 years) Improved self-efficacy (1 cohort)	No between-group differences in depression (3 RCTs) No significant differences in self-esteem (1 RCT and 2 non-RCTs) No significant effect on purpose in life (2) No significant effects for sense of usefulness (1 trial) No significant effects for sense loneliness (1 trial) No reduction in depression (2 cohort) No effect on life satisfaction (1 cohort) No effect on happiness (1 cohort)	17
Kragt & Holtrop (2019)	Volunteers were more extroverted, optimistic and perceived a greater sense of control in their lives compared to non-volunteers (1) Significant increase in mood states (1) Improved self-confidence when looking after patients with dementia (1)		− 15
Lovell et al. (2015)	Increase in mental health and well-being states (3)	No impact or significant improvement in mental health (1)	0
Manjunath & Manoj (2021)	Increased life satisfaction (1) Decreased likelihood of dementia treatment (1) Increased happiness (1)		− 11
Marco-Gardoqui et al. (2020)	Improved self-esteem and self-confidence (11) Increased motivation (4) Improved self-efficacy (3) Feeling of pride (2)		5
Milbourn et al. (2018)	Increase in psychological domain of quality of life (1) Significant increase in psychological quality of life when volunteering between one and 10 h of monthly (above that there was no effect) (1) Decreased depression (1) Slower decline in psychological well-being when volunteering under 100 h per year (1) Slower decline in mental health (1) Significantly increased life satisfaction when volunteering over 7 h weekly (1)	No increase in psychological well-being (combination of life satisfaction and mental health scores) compared to non-volunteers (1)	− 8
Onyx & Warburton (2003)	Improved self-esteem (1) Improved coping with stress (1) Improved adjustment to critical life events (2) Increased life satisfaction and decreased depression and anxiety (1)		
Owen et al., (2022)	Significant improvement in life satisfaction (1) Significant decrease in anxiety compared to active controls (1)	No significant decrease in depression compared to active controls (1)	5
Bonsdorff & Rantanen (2011)	Decreased depression (6 prospective) Significant increase in life satisfaction (1)		− 14
Wheeler et al (1998)	Significant increase in life satisfaction such that 70% of volunteers enjoy greater life satisfaction than the average non-volunteer. Adjusted for covariates reduced but did not diminish the effect		− 17
Willems et al. (2020)	High overall satisfaction (5) Feelings of altruism (2) Feeling useful (1) Increased purpose in life (1) Personal growth (1) Gratefulness (2)	3% of participants showed suicidal ideation (1) 22% of volunteers met criteria for a psychiatric diagnosis (1) More than 50% reported feeling burnout at some point (1) 77% showed symptoms of compassion fatigue (1) 46% scored high on disruptions of self-belief (1) Increased subjective distress (2) Increased post-shift stress (1)	1

The main effects of volunteering on psychological well-being clustered around those affecting mood and affect, and self-evaluations and concepts. For affect outcomes, reviews mostly reported a significant positive improvement in depression scores (Anderson et al., 2014; Bonsdorff & Rantanen 2011; Cattan et al., 2011; Filges et al., 2020; Giraudeau & Bailly, 2019; Höing et al., 2016; Onyx & Warburton, 2003). Only one review reported highly mixed findings (Jenkinson et al., 2013), possibly attributable to the higher quality of included primary studies (Jenkinson et al., 2013). Reviews reporting a smaller number of contributing studies found possible moderators; two reported a reduction in depression in women but not men (Anderson et al., 2014; Cattan et al., 2011), one found a reduction in older but not younger populations (Farrell & Bryant, 2009), and another found a reduction for general volunteering but increased depression for volunteering involving high empathetic arousal (Höing et al., 2016). In support of age as a moderator, the reviews finding a consistent positive effect on depression mainly focused on older adults (Bonsdorff & Rantanen 2011; Cattan et al., 2011; Filges et al., 2020), and the review with mixed findings included adults of all ages (Jenkinson et al., 2013).

There was more consistent evidence to support other mood and affect benefits, such as life satisfaction (Anderson et al., 2014; Cattan et al., 2011; Chen et al., 2022; Farrell & Bryant, 2009; Höing et al., 2016; Jenkinson et al., 2013; Manjunath & Manoj, 2021; Onyx & Warburton, 2003; Owen et al., 2022), positive affect (Anderson et al., 2014; Chen et al., 2022; Höing et al., 2016; Kragt & Holtrop, 2019; Manjunath & Manoj, 2021; Willems et al., 2020), and motivations (Goethem et al., (2014); Marco-Gardoqui et al., 2020), although a minority of evidence found non-significant effect of volunteering on life satisfaction (Anderson et al., 2014; Höing et al., 2016; Howard & Serviss, 2022; Jenkinson et al., 2013) and positive affect (Anderson et al., 2014; Jenkinson et al., 2013). The heterogeneity of findings is most likely attributable to all volunteering types being included (Anderson et al., 2014; Cattan et al., 2011; Farrell & Bryant, 2009; Höing et al., 2016; Jenkinson et al., 2013). Additionally, single reviews found a significant reduction in anxiety (Galbraith et al., 2015) and an increase in psychological well-being (Cattan et al., 2011). Although symptoms of burnout and emotional exhaustion was cited as a significant consequence of volunteering by one review (Höing et al., 2016), this included emotionally demanding volunteering roles including working with medium to high risk sex offenders.

Some reviews grouped prominent psychological benefits into self-evaluations or self-concepts (Conway et al., 2009; Goethem et al., (2014)). The most commonly reported effects on self-concepts were an increase in self-esteem (Anderson et al., 2014; Chen et al., 2022; Farrell & Bryant, 2009; Höing et al., 2016; Marco-Gardoqui et al., 2020; Onyx & Warburton, 2003), purposefulness, meaningfulness, satisfaction or accomplishment (Chen et al., 2022; Galbraith et al., 2015; Gualano et al., 2018; Höing et al., 2016; Willems et al., 2020), pride and empowerment (Farrell & Bryant, 2009; Giraudeau & Bailly, 2019; Höing et al., 2016; Marco-Gardoqui et al., 2020), and self-efficacy (Goethem et al., (2014); Marco-Gardoqui et al., 2020). However, there was some evidence of no significant effect on self-esteem (Anderson et al., 2014; Jenkinson et al., 2013) or purposefulness (Jenkinson et al., 2013).

#### Physical Effects on Health and Well-being

Outcomes relating to physical effects were the least commonly investigated, reported by only 13 reviews (Table 6). The most consistent positive effect on physical health was an increase in physical activity (Anderson et al., 2014; Bonsdorff & Rantanen 2011; Cattan et al., 2011; Chen et al., 2022; Lovell et al., 2015; Onyx & Warburton, 2003). Increased self-reported health (Anderson et al., 2014; Bonsdorff & Rantanen 2011; Cattan et al., 2011; Chen et al., 2022; Gualano et al., 2018; O’Flynn et al., 2021; Onyx & Warburton, 2003) and functional independence (Anderson et al., 2014; Cattan et al., 2011; Filges et al., 2020; Gualano et al., 2018; Höing et al., 2016) and reduced functional disability (Bonsdorff & Rantanen 2011; Höing et al., 2016; Milbourn et al., 2018) and mortality (Anderson et al., 2014; Bonsdorff & Rantanen 2011; Filges et al., 2020; Höing et al., 2016; Jenkinson et al., 2013; Okun et al., 2013; Onyx & Warburton, 2003) were also commonly cited benefits, although the evidence for these effects was more inconsistent (Anderson et al., 2014; Jenkinson et al., 2013). For example, there was evidence to suggest that benefits associated with self-reported health find a curvilinear relationship with intensity of volunteering, such that benefits only increase up until a moderate amount of hours spent volunteering (Anderson et al., 2014). The evidence for a decrease in mortality was the most substantial and, although reduced by the inclusion of covariates including SES, age, religious attendance, social support and health habits, remained significant (Jenkinson et al., 2013; Okun et al., 2013; Onyx & Warburton, 2003).

**Table 6:** Physical benefits

Review	Positive outcomes (number of studies)	Negative or non-significant outcomes	AMSTAR 2 rating
Anderson et al. (2014)	Increased self-reported general physical health (2 cross sectional, 2 descriptive, 6 prospective), curvilinear relationship (5 prospective) Maintenance of functional independence (8 prospective) Increased physical activity (3) Improved self-reported strength and walking speed (2) Less hypertension (1) (only in Caucasian Ps (1)) Fewer hip fractures (1) Reduction in mortality (13 prospective) (only for those who volunteered for other-oriented reasons (e.g. altruistic purposes) (1)) Reduction in mortality risk after adjusting for 14 covariates (1)	No association with general physical health (2 descriptive) (brief scales) Mixed results for grip strength (2) No association with physician-diagnosed medical conditions (3) No association with admission to a nursing home (1) No relation to mortality (2)	− 10
Cattan et al. (2011)	Improved self-rated health (1) Improved self-rated health/mental health (8) Improved physical/mental health (7) Improved functional status (4) Increased physical activity (3)		
Chen et al. (2020)	Increased physical activity (4) Increased perceived health (2) Reduction in laziness (1) Improved strength (1) and grip strength (1) Improved flexibility, (1) Improved mobility (2) Reduced blood pressure (2)	No significant improvement in BMI, cholesterol, LDL, TG, blood sugar, CRP, or cortisol (1)	9
Filges et al. (2020)	Reduction in mortality (all reported results) Reduced functional disability (all reported results) Increased Instrumental activities of daily living (IADL) (2) Improved maintenance of functional competence (all reported results)		30
Gualano et al. (2018)	Significant improvement in functional abilities (1) Significant increase in self-reported health (1)		9
Höing et al. (2016)	Increased maintenance of good health (12) Delayed onset of serious illness and functional disability (2) Reduction in mortality (6)	Did not improve bad health (12)	− 7
Jenkinson et al. (2013)	Increased physical activity (1) Increased strength (1) Significant reduction in mortality (4 cohort) Significant reduction in mortality when adjusting for covariates (5 cohort) Increased self-rated health (2 cohort)	No significant effect on number of falls (1) No significant effect on cane use (1) Inconclusive evidence for effect on functional abilities (3 cohort) No association with frailty (1) No association with chronic conditions (1) No difference in self-rated health (1 RCT) No association of mortality with volunteering (3 cohort) No effect on self-rated health (1), only for environmental volunteering (1)	17
Lovell et al. (2015)	Increased grip strength (1) Significant increase in self-reported physical activity (3)	No significant improvements in aerobic capacity, BMI, weight, body composition, flexibility, blood pressure, balance or hip/waist ratio (1)	0
Milbourn et al. (2018)	Increase in physical domain of quality of life (1) Weakened the association between age and functional decline (1) Increased survival and self-perceived health benefits when combined with paid employment (1)	No significant differences in risk of accumulating chronic medical conditions (1)	− 8
O’Flynn et al. (2021)	55% of respondents reported health as ‘increased’ or ‘increased greatly’ (1)		
Okun et al. (2013)	Reduced mortality by almost 50% (25), decreased to around 25% when adjusting for covariates		− 9
Onyx & Warburton (2003)	Reduction in mortality (2), reduced by sustained when controlling for covariates (2) Predicted positive health outcomes 30 years later (1) Reduced smoking and increased exercise (4) Reduced risk of institutionalisation (1) Increase in perceived health (6 cross sectional, 1 longitudinal) Increase in life satisfaction (1 longitudinal)		
Bonsdorff & Rantanen (2011)	Improved self-rated health (5) Reduced disability in activities of daily living tasks (5) Lower levels of functional dependency (1 longitudinal) Increased physical activity (3) Positive trend towards improved physical functioning (1) Reduced mortality in older adults (5 prospective)	No association with number of self-reported physician-diagnosed chronic diseases (2) No prediction of living at a nursing home 7 years later (1)	− 14

Evidence for improvements in blood pressure (Chen et al., 2022; Lovell et al., 2015) and grip strength (Anderson et al., 2014; Chen et al., 2022; Lovell et al., 2015) was sparse and inconsistent. There was no evidence for volunteering as a significant predictor of number of medical conditions (Anderson et al., 2014; Bonsdorff & Rantanen 2011; Milbourn et al., 2018), BMI (Chen et al., 2022; Lovell et al., 2015), frailty (Anderson et al., 2014; Jenkinson et al., 2013), or living in a nursing home (Anderson et al., 2014; Bonsdorff & Rantanen 2011). One review concluded that whilst volunteering helped to maintain good health, it did not improve bad health (Höing et al., 2016). Only one review reported decreased smoking (Onyx & Warburton, 2003).

#### Social Effects on Health and Well-being

A total of 15 reviews reported social outcomes from volunteering (Table 7). When social support, sense of community and social network were combined, the evidence mostly found volunteering to improve social outcomes (Anderson et al., 2014; Cattan et al., 2011). Individually, there was evidence in support of volunteering increasing social integration (Lovell et al., 2015; Marco-Gardoqui et al., 2020), but most commonly social network (Blais et al., 2017; Farrell & Bryant, 2009; Gualano et al., 2018; Höing et al., 2016), and social connectedness or a sense of community (Chen et al., 2022; Kragt & Holtrop, 2019; O’Flynn et al., 1971; Willems et al., 2020), with only a minority of evidence indicating no significant effect of volunteering in increasing one’s social network (Anderson et al., 2014). Volunteering was found to increase social support from both other volunteers (Höing et al., 2016) and friends and neighbours (Milbourn et al., 2018). There also appeared to be some knock-on effects, as an increased number of friendships in turn increased social integration (Farrell & Bryant, 2009) and increased social connectedness increased motivations (Willems et al., 2020). Only one review reported a negative effect, namely that whilst the number of positive social ties were increased, so were the number of negative social ties (Milbourn et al., 2018). Another caveat reported was that although social ties was beneficial, less than half of volunteers reported forming connection with volunteers (Hyde et al., 2014).

**Table 7:** Social Benefits

Review	Positive outcomes (number of studies)	Negative or non-significant outcomes	AMSTAR 2 rating
Anderson et al. (2014)	Social support/network (7 descriptive, 2 cross-sectional)	No association with social network (1 descriptive, 1cross-sectional)	− 10
Blais et al. (2017)	Built relationships and friendships (1) New-found friendships motivated continued volunteering (1)		− 18
Cattan et al. (2011)	Social networks/support/integration (6) Social/human/cultural capital (1) Social productivity/contrib. to organisation (6)		
Chen et al. (2020)	Reduced isolation (1) Increased social interaction (1) Improved compassion for others (1) Increased social connectivity (3)		9
Farrell & Bryant (2009)	Improved social integration and well-being (4) Increased opportunities for social engagement (1) Increased social networks for people with mental health problems (2)		− 15
Goethem et al., (2014)	Small but significant effect on social competence (social efficacy, abilities, skills) (23)		− 4
Gualano et al. (2018)	Significant maintenance of intergenerational interactions (1)		9
Höing et al. (2016)	Increased social support and interaction (1) Improved quantity and quality of social networks (5) Improved feelings of connectedness (2) Enjoyment of receiving support from other volunteers (4) Increased sense of belonging (1) Increased emotional attachment to others (1)		− 7
Hyde et al., (2014)	Appreciation from staff and families (1) Increase in social ties (1)	Only 44.6% of volunteers reported forming close social connections with other volunteers (1)	− 2
Kragt & Holtrop (2019)	Improved social well-being (1) Increased social connectedness (3) Forming relationships (1) Increased sense of community (1)		− 15
Lovell et al. (2015)	Increased social function (1)		0
Marco-Gardoqui et al. (2020)	Greater social engagement (most cited outcome)		5
Milbourn et al. (2018)	Increase in social domain of quality of life (1) Increase in social support from friends and neighbours (2) Increase in positive exchanges and social ties (2)	Volunteering predicted negative social ties (1)	− 8
O’Flynn et al. (2021)	Increased sense of community (4) Increase in valued relationships (1)		− 14
Willems et al. (2020)	Increased connectedness (2) (which in turn increased motivation (3))		1

#### Moderators and Mediators on the Effects on Health and Well-being

Several moderators were explored around the aspects of volunteering. Evidence for the most beneficial frequency of volunteering was mixed; whilst some reviews reported a positive linear relationship between volunteering frequency and benefits (Cattan et al., 2011; Goethem et al., (2014); Höing et al., 2016), others including the best quality evidence to report on optimal frequency (Jenkinson et al., 2013) reported inconsistent findings (Anderson et al., 2014; Cattan et al., 2011; Jenkinson et al., 2013; Okun et al., 2013). Some reviews reported a curvilinear relationship between frequency and benefits (Conway et al., 2009; Höing et al., 2016; Milbourn et al., 2018; Onyx & Warburton, 2003), such that a moderate intensity of volunteering maximised the benefits, although these reviews were poor quality. The suggested optimal intensity was suggested to be around 2 h per week or 100 h per year (Anderson et al., 2014; Höing et al., 2016; Milbourn et al., 2018). There was disagreement as to whether formal volunteering is more (Cattan et al., 2011; Conway et al., 2009; Wheeler et al., 1998) or less (Cattan et al., 2011; Hui et al., 2020) beneficial than informal volunteering. This was possibly due to the outcome measure, as direct formal volunteering significantly increased life satisfaction (Wheeler et al., 1998), whilst mixed or informal helping significantly increased well-being and psychological functioning compared to formal volunteering (Hui et al., 2020). One review focusing on adolescents found no moderation of type of volunteering (Goethem et al., (2014)), but another higher quality review reported only beneficial effects of environmental volunteering on physical health in comparison to civic volunteering (Jenkinson et al., 2013). In contrast, there was consistent evidence that structured reflection was an important positive predictor of health outcomes (Conway et al., 2009; Goethem et al., (2014)). Religious volunteering was also a consistently reported moderator for positive health benefits (Bonsdorff & Rantanen 2011; Höing et al., 2016; Manjunath & Manoj, 2021; Okun et al., 2013), with one review finding a partially mediating role of volunteering on the beneficial effects of religiosity on well-being (Kragt & Holtrop, 2019).

Several factors were explored in relation to the characteristics of the volunteer. Age was the most consistently reported demographic factor as a significant moderator of the effects of volunteering on well-being. Generally, older age predicted larger effects on positive health outcomes (Anderson et al., 2014; Goethem et al., (2014); Gualano et al., 2018; Höing et al., 2016; Jenkinson et al., 2013), and there was inconsistent evidence to suggest these increased effects were related to retirement (Höing et al., 2016; Hui et al., 2020). Whilst one review reported older adults volunteering to experience greater satisfaction than older adults in employment (Kragt & Holtrop, 2019), another higher quality review found older adults both working and in employment saw the most beneficial effects on health and well-being (Milbourn et al., 2018). On the other hand, younger age predicted higher emotional exhaustion and distress in emotionally demanding volunteering roles such as crisis line, with positive coping strategies and organisational support key to reducing this (Willems et al., 2020). There was minimal evidence of gender as a moderator of volunteering and well-being (Okun et al., 2013), with mostly no effect found (Goethem et al., 2014; Hui et al., 2020). The issue of self-selection was frequently discussed. Some reviews reported that those of higher SES were more likely to volunteer, creating a sampling bias in the results (Bonsdorff & Rantanen 2011; Cattan et al., 2011). However, the effect of volunteering on mortality was reduced but still significant when adjusting for covariates such as SES (Okun et al., 2013). Also, there was some evidence to suggest that those of lower SES felt more empowered by volunteering (Cattan et al., 2011) and reported more health benefits (Cattan et al., 2011; Höing et al., 2016). However, higher education was found to decrease stress when volunteering for crisis line (Willems et al., 2020).

Motivations for volunteering was found to be a significant moderator, such that those with altruistic or intrinsic motivations for volunteering saw increased benefits than those motivated for other reasons (Anderson et al., 2014; Höing et al., 2016; Okun et al., 2013). In support, one review found prosociality to be a far stronger predictor of health and well-being than volunteering alone (Hui et al., 2020). Feeling appreciated was found to be necessary to see improvements in quality of life (Jenkinson et al., 2013) or moderated the effects (Anderson et al., 2014). A moderating effect of feeling appreciated on health outcomes was also reported for depression, life satisfaction, and general well-being (Anderson et al., 2014). Although empathising with the recipient was important for spiritual development, it also increased the likelihood of burnout in emotionally demanding volunteering roles (Willems et al., 2020).

Some interactions were explored between the effects. The most frequently discussed was social factors including social connection, support, and interaction, which often moderated the relationship between volunteering and other health outcomes (Höing et al., 2016; Milbourn et al., 2018; Okun et al., 2013; Onyx & Warburton, 2003), with one review finding them to be a complete mediator of volunteering and life satisfaction (Anderson et al., 2014). For emotionally demanding volunteering such as crisis line, social support helped to increase well-being and buffer any negative effects (Wheeler et al., 1998). In keeping with this, one review hypothesised that volunteering generates social capital for both the recipient and the volunteer, with subsequent benefits on health and well-being (Onyx & Warburton, 2003).

#### Findings from Meta-Analyses

Results from reported meta-analyses (Table 8) varied on measures used to calculate both pooled estimates and heterogeneity, meaning comparison between reviews was difficult. There was also a lack of reporting heterogeneity at all, reflecting the general poor quality of included reviews. There were no available meta-analyses for social outcomes, aside from an aggregate measure of personal and social competence. Although many were significant, the pooled estimates for most outcomes were small, aside from mortality (Filges et al., 2020; Okun et al., 2013), and measures of physical functionality such as maintenance of functional competence (Filges et al., 2020). Mortality (Filges et al., 2020; Okun et al., 2013) and well-being (Conway et al., 2009; Howard & Serviss, 2022; Hui et al., 2020) were the only two outcomes reported by meta-analyses of more than one review. For both outcomes, pooled estimates were similar across reviews.

**Table 8:** Table of meta-analyses

Coding of outcome	Outcome	Volunteering type	Review	No. included studies	Heterogeneity (default: I squared)	CI	Pooled estimate	AMSTAR 2 rating
Psychological	Self-evaluations	Service learning	Conway et al. (2009)	32	(true standard deviation of difference) .25	.16–.37	(mean difference in means) .26	− 20
	Attitudes towards the self	General (adolescents)	Goethem et al., (2014)	15	(fail safe number) 11	.04–.69	.36	− 4
	Life satisfaction (unadjusted)	General (older adults)	Wheeler et al (1998)	29	No statistic reported	.19–.31	.25	− 17
	Job satisfaction	Employee-level participation	Howard & Serviss (2022)	7	No statistic reported	–.02–.15	(sample size weighted average correlation) .06	− 17
		Organisational-level participation	Howard & Serviss (2022)	4	No statistic reported	.24–.37	(sample size weighted average correlation) .31	− 17
	Psychological functioning	Volunteering/helping (frequency)	Hui et al., (2020)	53	No statistic reported	.09–.14	.12	− 12
		Volunteering/helping (binary)	Hui et al., (2020)	72	No statistic reported	.11–.16	.14	− 12
		Formal volunteering	Hui et al., (2020)	108	No statistic reported	.10–.13	.12	− 12
	Psychological malfunctioning	Volunteering/helping (frequency)	Hui et al., (2020)	35	No statistic reported	.03–.12	.07	− 12
		Volunteering/helping (binary)	Hui et al., (2020)	30	No statistic reported	.08–.20	.14	− 12
		Formal volunteering	Hui et al., (2020)	55	No statistic reported	.07–.13	.11	− 12
	Depression severity	General (older adults)	Filges et al. (2020)	3	12%	.00–.23	.12	30
Physical	Mortality (unadjusted)	Organisational volunteering	Okun et al. (2013)	25	82%	.45–.62	.53	-9
	Mortality (adjusted*)		Okun et al. (2013)	11	59%	.69–.84	.76	− 9
	Mortality (HR)	General (older adults)	Filges et al. (2020)	8	0%	.72–.80	.76	30
	Mortality (OR)		Filges et al. (2020)	2	0%	.58–.83	.69	30
	Incident functional disability		Filges et al. (2020)	3	27%	.72–.97	.83	30
	Instrumental activities of daily life		Filges et al. (2020)	2	0%	.53–1.01	.73	30
	Maintenance of functional competence		Filges et al. (2020)	3	0%	.70–.94	.81	30
	Physical health	Volunteering/helping (frequency)	Hui et al., (2020)	33	No statistic reported	.06–.09	.08	− 12
		Volunteering/helping (binary)	Hui et al., (2020)	36	No statistic reported	.08–.20	.10	− 12
		Formal volunteering	Hui et al., (2020)	74	No statistic reported	.07–.13	.09	− 12
General	Well-being	Service learning	Conway et al. (2009)	6	(true standard deviation of difference) .26	− .07–.42	(mean difference in means) .17	− 20
		Employee-level participation	Howard & Serviss (2022)	4	No statistic reported	− .12–.51	(sample size weighted average correlation) .22	− 17
		Organisational-level participation	Howard & Serviss (2022)	3	No statistic reported	.11–.36	(sample size weighted average correlation) .24	− 17
		Volunteering/helping (frequency)	Hui et al., (2020)	56	No statistic reported	.07–.13	.10	− 12
		Volunteering/helping (binary)	Hui et al., (2020)	75	No statistic reported	.11–.16	.14	− 12
		Formal helping	Hui et al., (2020)	111	No statistic reported	.09–.13	.11	− 12
		Informal helping	Hui et al., (2020)	61	No statistic reported	.12–.18	.15	− 12
Mixed	Personal and social competence	General (adolescents)	Goethem et al., (2014)	23	(fail safe number) 76	.11–.39	.25	− 4

*Adjusted mortality controlled for covariates including age, sex, ethnicity, socioeconomic status, work status, marital status, religiosity, emotional health, health behaviours, social connection, social interaction, and physical health

## Discussion

**Fig. 3:**
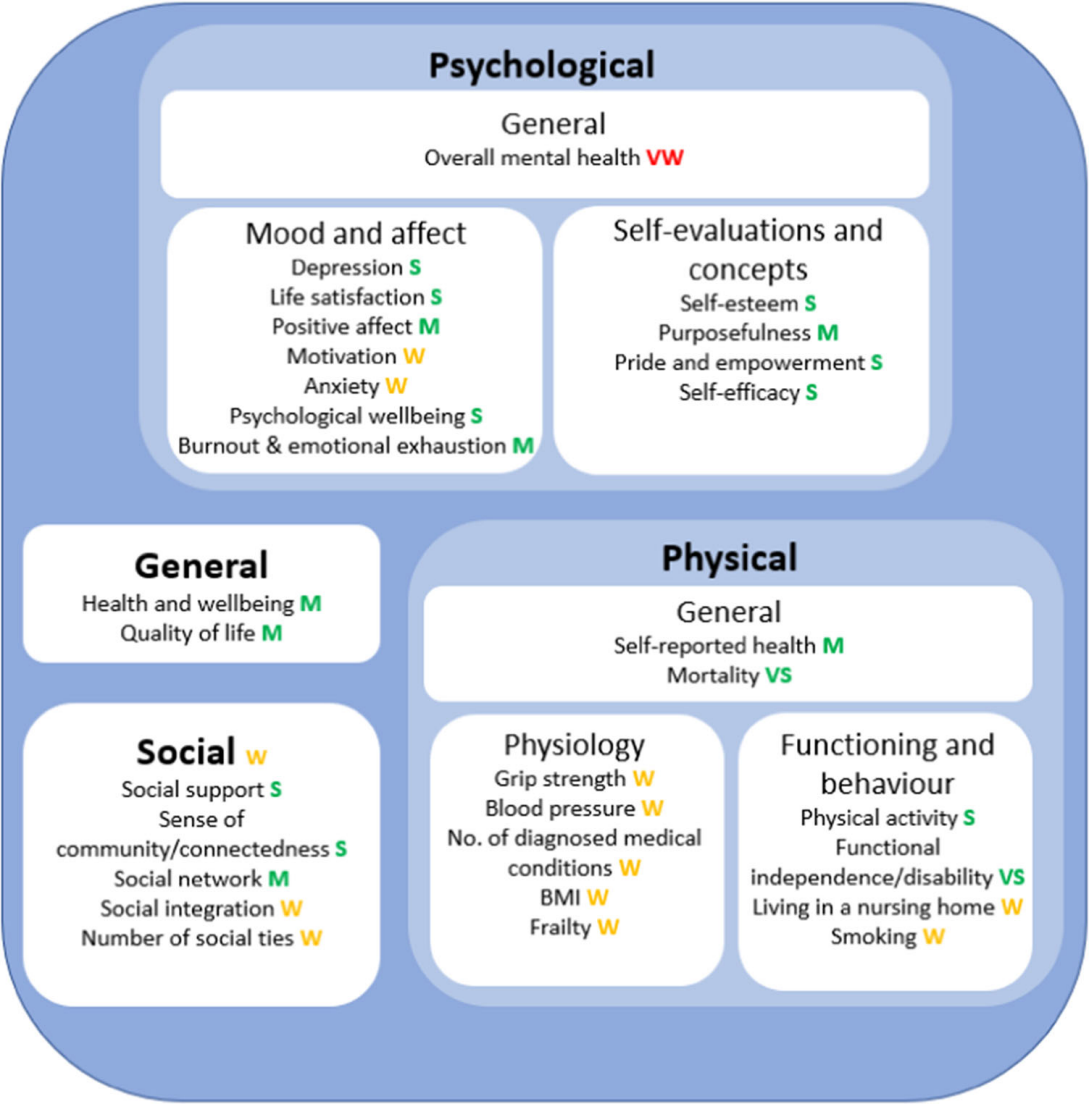
Summary of strength of evidence for each variable outlined in Fig. 1. Labelled according to vote counting results; ‘very strong’, ‘strong’, ‘moderate’, ‘weak’, and ‘very weak’

The current umbrella review identified 28 eligible reviews, mostly focusing on older adults, based in the USA, and including a range of forms volunteering. An overview of the strength of the evidence for each variable is shown in Fig. 3. Reduced mortality and improved physical functioning showed the largest effect sizes with consistent supporting evidence. There was also consistent evidence to support effects on general health and well-being and quality of life, psychological well-being, pride and empowerment, motivation, self-efficacy, life satisfaction, positive affect, reduced depression, and purposefulness related to psychological constructs, improved self-reported health and physical activity relating to physical benefits, and improved social support, sense of connectedness and community, and network. The evidence suggests no effect of volunteering on medical conditions, BMI, frailty, or living in a nursing home. More research is required to establish whether there are effects of volunteering on blood pressure and grip strength. Organisational-level participation, older age, reflection, religious volunteering, altruistic motivations, and feeling appreciated all amplify the relationship between volunteering and health and well-being. Additionally, social factors have a knock-on effect for other health and well-being outcomes, with protective effects for any potential negative outcomes. There was no evidence of moderation of gender. More research is needed to explore the optimal intensity of volunteering, the role of SES, whether formal or informal volunteering is most beneficial, and whether the moderation of age is related to retirement, as current evidence is inconsistent.

Age was the most supported moderator, namely that those of older age received greater health benefits from volunteering. One reason is that volunteering compensates for the loss of the health and well-being benefits of career success (Spurk et al., 2019), easing the adjustment to retirement. In support of this, work related satisfaction and perceived rewards significantly predicted life satisfaction in retired volunteers, even when controlling for demographic factors and self-efficacy (Wu et al., 2005). However, the current umbrella review found inconsistent evidence to support retirement as the explanation. Instead, the findings indicate that although many of the benefits associated with volunteering do relate to a sense of purpose, the benefits of volunteering are also distinct from usual work activity, through feelings of altruism and self-actualisation. This perhaps explains the complex relationship with age. Age has been established as a positive predictor of altruistic motivations (Sparrow et al., 2021), which was found to predict better health outcomes of volunteering. More research is needed to explore the role of retirement and alternate explanations in the relationship between age and the benefits of volunteering, including the interaction of age with other moderators.

On the contrary, there was no evidence to support gender as a moderator for the relationship between volunteering and health and well-being. Although women are more likely to volunteer than men (NCVO, 2021b), the results of this review indicated that once volunteering, there is no effect of gender on the subsequent health benefits. This provides a case for future volunteering initiatives to be targeted towards men, and for more research to explore the barriers to volunteering for men specifically, such as through qualitative methodology (Males, 2015).

The findings of this review suggest a complex relationship between SES and volunteering and its benefits. There is vast research to support the finding that those of higher SES are twice as likely to volunteer than those of the lowest SES (NCVO, 2021b). However, the current review also indicated that those of lower SES may benefit more from volunteering. If so, the use of volunteering must be maximised to help reduce health inequalities. It is key to note that those of lower SES are more likely to engage in informal volunteering, which is often overlooked by the volunteering literature (Dean, 2022). Thus, it is important that future research further explore the influence of the formality of volunteering on the health benefits, as the current umbrella review found inconsistent results. Dependent on this, particularly during retirement, the findings of this review indicate that public health campaigns to enable volunteering should be particularly focused on those of lower SES.

More research is needed to determine the relationship between frequency of volunteering and health and well-being, as the current review found it was not related to the age of volunteers or type of volunteering. The rationale behind a curvilinear relationship is that time spent volunteering positively predicts burnout (Moreno-Jiménez & Villodres, 2010). However, the only evidence linking volunteering to burnout in the current umbrella review related to volunteering that was emotionally demanding (Höing et al., 2016; Willems et al., 2020) rather than frequency, as suggested by Linning and Jackson (Linning & Volunteering, 2018). Indeed, emotional exhaustion is one of three subscales within the concept of burnout, which is explained as a result of prolonged and intense emotional involvement (Maslach & Jackson, 1981). The current umbrella review found that sufficient support from the organisation helped mitigate the effects of emotionally demanding volunteer roles on burnout and increased well-being (Höing et al., 2016; Kragt & Holtrop, 2019; Willems et al., 2020). Systematic reviews of healthcare providers have found a negative prediction of positive social support to burnout, leading the authors to recommend that interventions to reduce burnout should focus on social support (Guilaran et al., 2018; Velando-Soriano et al., 2020). Thus, it is at upmost importance that organisations recruiting for emotionally demanding volunteer roles must ensure a sufficient and positive support network to avoid negative health and well-being outcomes such as burnout. For example, sufficient support from supervisors and a stable and supportive organisational environment are essential.

A particularly useful finding of this review is that positive social outcomes of volunteering in turn encourage other positive health and well-being outcomes. Indeed, social capital has been established to reduce mortality and improve physical and mental health (Ehsan et al., 2019). Interestingly, the current review also found that volunteering predicted self-reported health, functioning, mortality, and mental health outcomes much better than for other objective indicators of health such as living with medical conditions, BMI, and frailty. This highlights the need for a holistic view of health to assess mortality risk rather than only focusing on physical indicators. For example, lack of flourishing mental health was shown to significantly predict mortality in a 10-year longitudinal analysis, even when controlling for a number of factors including physical disease (Keyes & Simoes, 2012). Another longitudinal study found that although the prediction of life satisfaction on mortality was partially shared with physical health and social orientation, it also exerted an independent effect on mortality (Hülür et al., 2017). Thus, it is essential to also focus on the mental and social outcomes of volunteering to capture all the potential benefits.

There was consistent evidence to suggest religious volunteering to be a moderator of the effects of volunteering on health and well-being. Whilst one suggested explanation for the moderating effect on well-being is that religiosity is an indication of benevolent and altruistic motives (Krause et al., 2017), the social science literature suggests that volunteering offers a chance to enact a group identity (Caricati et al., 2020; Gray & Stevenson, 2020), in this case a religious group (Wakefield et al., 2022). Indeed, for volunteers high in religiosity, identification with the religious organisation they were volunteering for predicted their sense of being enable to enact their religious group three months later, which in turn predicted mental health improvements (Wakefield et al., 2022). Subsequently, the relationship between religion, volunteering and well-being is not only explained through altruistic motives, but also because volunteering provides those high in religiosity a space to enact their religious norms, strengthening their group identity and consequently their well-being (Wakefield et al., 2022). However, more research is needed to determine whether this also applies when volunteering for secular organisations.

### Strengths and Limitations

The current umbrella review provides a comprehensive overview of the literature on the benefits of all types of volunteering (Gianfredi et al., 2022). Furthermore, the very low overlap of primary studies provides credibility to the conclusions drawn. However, there are a number of limitations to consider. The relatively high proportion of articles retrieved from other sources, despite scoping searches being conducted prior to the search, indicates that the databases searched were not comprehensive. Forward and backward citation searching aimed to address this limitation. Secondly, the included reviews were mainly low quality, and for those reviews that assessed quality, the quality of primary studies was mixed. However, as higher quality reviews tended to use a more stringent measure of risk of bias (Chen et al., 2022; Filges et al., 2020; Gualano et al., 2018; Jenkinson et al., 2013), it is important that the quality of the review was also considered when weighting findings. Whilst the very low percentage of overlap between primary was a strength, it also may indicate that the included reviews were not thorough, reflected in the general poor quality ratings. Also, the vote counting method applied could not account for the curvilinear relationships identified, highlighting the importance of describing these within the text. More significantly, although efforts were made to conduct vote counting via direction of effect rather than significance, this was not always possible to attain due to insufficient reporting of reviews.

Another limitation is that although three reviews were published in 2022, none of the searches went beyond 2020, meaning no research conducted during or after the COVID-19 pandemic was included. There is evidence that the COVID-19 pandemic created lasting changes to volunteering, mainly that it encouraged digital volunteering which has sustained even after restrictions were lifted (Kanemura et al., 2022). This digitalisation has attracted a new group of volunteers who may experience volunteering differently (Kanemura et al., 2022). More importantly, digitalisation has impacted on the opportunity for social connection (Kanemura et al., 2022), which, as established by this review, has a knock-on effect on the mental and physical benefits of volunteering. A systematic review of research conducted after 2020 would be useful to compare to the findings of the current umbrella review to explore these differences further.

## Conclusion

This review has established a multitude of benefits of volunteering on mental, physical, and social health and well-being, particularly reduced mortality, and increased functioning, quality of life, pride, empowerment, motivation, social support, and sense of community. To ensure the generalisability of these findings, more research is needed outside of the USA, and specifically focusing on adolescents. More quantitative research to aid meta-analyses on the social benefits of volunteering would be beneficial to quantify the effects and aid comparison with the mental and physical benefits. However, any future systematic review and meta-analysis on the topic should ensure to follow quality criteria from the AMSTAR-2 (Shea et al., 2021), specifically ensuring to pre-register methods and hypotheses, cite excluded studies, report their funding source, and account for their risk of bias. Concerning interacting factors, more research is needed to explore the likely complex relationship of volunteering with both SES and religiosity, and the optimum ‘dose’ of volunteering to gain the established benefits. Volunteering should be considered as an intervention in itself, particularly within the context of social prescribing, where referral to engage in volunteering should be encouraged. Where volunteering roles are emotionally demanding, an appropriate support system should be ensured by the organisation to prevent negative health outcomes such as burnout.

## Supplementary Information

Below is the link to the electronic supplementary material.

Supplementary file1 (DOCX 226 KB)
