# Supplementary material for: Exploring the Effects of Volunteering on the Social, Mental, and Physical Health and Well-being of Volunteers: An Umbrella Review
Source: Voluntas. 2023 May 4:1–32. Online ahead of print. doi: 10.1007/s11266-023-00573-z (PMC10159229; doi:10.1007/s11266-023-00573-z)
Supplement: Supplementary file 1 — Supplementary file1 (DOCX 226 KB) [file 11266_2023_573_MOESM1_ESM.docx]

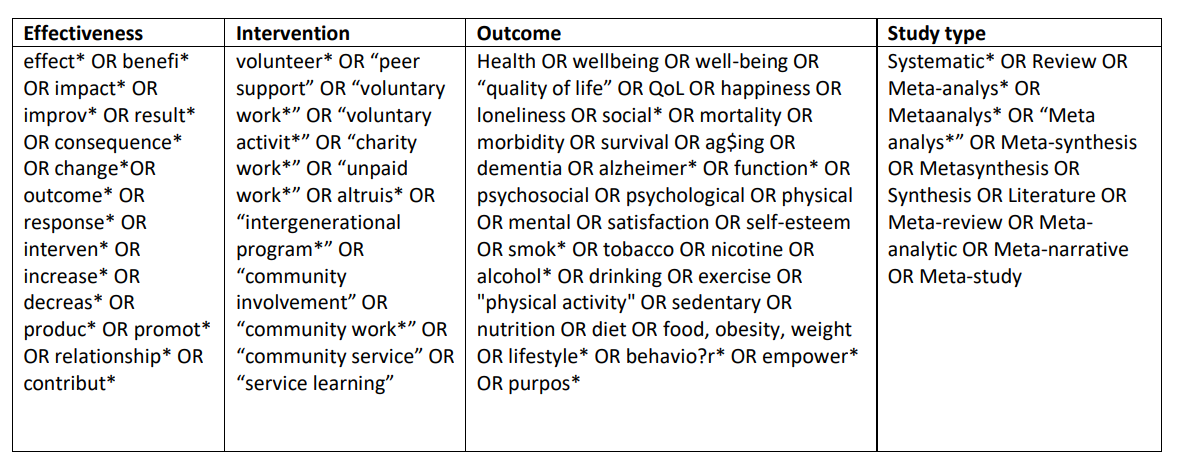
Supplementary material 1: Search strategy

Supplementary Material 2: Inter-rater reliability

At both stages, second reviewer CH independently screened the first ten percent of articles, sorted alphabetically by author. All decisions were made and logged via Rayyan. To measure inter-rater reliability, Cohen’s Kappa statistic (33) was calculated at both stages, applying the conservative parameters by Altman (34). Additionally, any disagreements between reviewers were resolved by discussion until a consensus was reached.

Supplementary material 3: Quality appraisal

To ensure a comprehensive synthesis, no studies were excluded based on quality (1). Instead, quality was considered when synthesising and discussing results. Although AMSTAR 2 is not intended to produce a score or rating (2), a scoring system was applied to aid in the weighting of results. Similar to scoring applied by Kilich et al. (3), items marked ‘yes’ were assigned two points and ‘partial yes’ assigned one point, and items marked ‘no’ deducted two points from the total score. If the item was not applicable (i.e no meta-analysis was conducted), the item was not scored. Consequently, the minimum and maximum scoring was minus 32 and plus 32, respectively. Again, reviewer CH assessed 10% of studies for quality, Cohen’s Kappa statistic calculated, and any disagreements were resolved through discussion.

To assess quality of the primary included studies, quality appraisal by the review was used. Of the reviews which assessed quality, the quality appraisal for the primary studies was presented alongside each other below the quality appraisal for the review, as a visual aid to compare when reviews included the same primary study.

Supplementary material 4: Data extraction

As with article screening and quality appraisal reviewer CH extracted data from the first 10% of included reviews and any disagreements were resolved through discussion. Three authors were contacted for necessary information for the overlap table. Data was only extracted for primary data that contributed to the research question. For example, many of the included studies of the reviews on intergenerational programmes involved activities that could not be classified as volunteering for either generation. It also varied as to who was considered the volunteer and who the recipient, with some involved in volunteering together. Consequently, results were only extracted for the eligible studies and for the relevant outcomes.

Supplementary material 5: Excluded articles

The main reason for exclusion of articles based on full texts was that they did not describe a systematic review (4-22). The remaining reasons were that the outcome was not health benefits (23-29), articles did not meet the definition of volunteering outlined for this review (30-34), outcomes were concerning benefits for the recipient (35-38), the article was not peer-reviewed (39, 40), results were triangulated with qualitative data (41), the review was of qualitative or mostly qualitative literature (42, 43), the abstract was for an oral presentation (44, 45), the review was not published in English (46), and the review had already been included as a different paper (47).

Supplementary material 6: Inter-rater agreement (*reviewers blinded for referees*)

As ‘maybe’ was used during study inclusion based on title and abstract by one reviewer (CH), the kappa statistic was calculated using three variables, and was found to be very good (99.43%). Inter-rater agreement was also very good for screening based on full text (100%), and good for quality appraisal (73%). Again, the kappa statistic for quality appraisal was calculated using three responses, and responses marked ‘no-meta analysis conducted’ were not included in the calculation.

References

1. Gianfredi V, Nucci D, Amerio A, Signorelli C, Odone A, Dinu M. What Can We Expect from an Umbrella Review? Advances in Nutrition. 2022;13(2):684-5.

2. Shea BJ, Reeves BC, Wells G, Thuku M, Hamel C, Moran J, et al. AMSTAR 2: a critical appraisal tool for systematic reviews that include randomised or non-randomised studies of healthcare interventions, or both. bmj. 2017;358.

3. Kilich E, Dada S, Francis MR, Tazare J, Chico RM, Paterson P, et al. Factors that influence vaccination decision-making among pregnant women: A systematic review and meta-analysis. PloS one. 2020;15(7):e0234827.

4. Rosenkranz RR. Service-learning in Higher Education Relevant to the Promotion of Physical Activity, Healthful Eating, and Prevention of Obesity. International Journal of Preventive Medicine. 2012;3(10).

5. Cipriani J. Altruistic Activities of Older Adults Living in Long Term Care Facilities: A Literature Review. Physical and Occupational Therapyin Geriatrics. 2007;26(1):19-28.

6. Miller KD, Schleien SJ, Brooke P, Frisoli AM, Brooks WT, III. Community for All: The Therapeutic Recreation Practitioner's Role in Inclusive Volunteering. Therapeutic Recreation Journal. 2005;39(1):18-31.

7. Drebing CE, Reilly E, Henze KT, Kelly M, Russo A, Smolinsky J, et al. Using peer support groups to enhance community integration of veterans in transition. Psychological Services. 2018;15(2):135-45.

8. Stukas AA, Snyder M, Clary EG. Understanding and encouraging volunteerism and community involvement. The Journal of Social Psychology. 2016;156(3):243-55.

9. Morrow-Howe N. Volunteering in Later Life: Research Frontiers. The Journals of Gerontology. 2010;65(4):461.

10. Gottlieb BH, Gillespie AA. Volunteerism, health, and civic engagement among older adults. Canadian journal on aging = La revue canadienne du vieillissement. 2008;27(4):399-406.

11. Wilson J. Volunteerism Research: A Review Essay. Nonprofit and Voluntary Sector Quarterly. 2012;41(2):176-212.

12. Reeb RN, Folger SF, Langsner S, Ryan C, Crouse J. Self-Efficacy in Service-Learning Community Action Research: Theory, Research, and Practice. American Journal of Community Psychology. 2010;46(3):459-71.

13. Champagne N. Service Learning: Its origin, evolution, and connection to health education*. American Journal of Health Education. 2006;37(2):97-102.

14. Omoto AM, Snyder M. Considerations of community: the context and process of volunteerism. American Behavioral Scientist. 2002;45(5):846-67.

15. Kuperminc GP, PhD, Holditch PT, BA, Allen JP, PhD. Volunteering and community service in adolescence. Adolescent Medicine. 2001;12(3):445-IX.

16. Lavelle JJ. What motivates OCB? Insights from the volunteerism literature. Journal of Organizational Behavior. 2010;31(6):918.

17. Claxton-Oldfield S. Hospice palliative care volunteers: The benefits for patients, family caregivers, and the volunteers. Palliative & Supportive Care. 2015;13(3):809-13.

18. Holmgren C, Benzian H. Dental volunteering - a time for reflection and a time for change. British Dental Journal. 2011;210(11):513-6.

19. Moore CW, Allen JP. The effects of volunteering on the young volunteer. The journal of primary prevention. 1996;17(2):231-58.

20. Ballard PJ, Daniel SS, Anderson G, Nicolotti L, Caballero Quinones E, Lee M, et al. Incorporating Volunteering Into Treatment for Depression Among Adolescents: Developmental and Clinical Considerations. Frontiers in psychology. 2021;12:642910.

21. Serrat R, PhD, Scharf T, PhD, Villar F, PhD, Msc CG. Fifty-Five Years of Research Into Older People's Civic Participation: Recent Trends, Future Directions. The Gerontologist. 2020;60(1).

22. Lucchetti G, Granero Lucchetti AL, Bassi RM, Nobre MRS. Complementary Spiritist Therapy: Systematic Review of Scientific Evidence. Evidence - Based Complementary and Alternative Medicine. 2011;2011.

23. Jones FA, Knights DP, Sinclair VF, Baraitser P. Do health partnerships with organisations in lower income countries benefit the UK partner? A review of the literature. Globalization and Health. 2013;9:-38.

24. Leger J, Letourneau N. New mothers and postpartum depression: a narrative review of peer support intervention studies. Health & Social Care in the Community. 2015;23(4):337-48.

25. Toner S, Hickling LM, Costa MPd, Cassidy M, Priebe S. Characteristics, motivations and experiences of volunteer befrienders for people with mental illness: a systematic review and narrative synthesis. BMC Psychiatry. 2018;18.

26. Whittall D, Lee S, O'Connor M. Factors affecting rural volunteering in palliative care - an integrated review. Australian Journal of Rural Health. 2016;24(6):350-6.

27. Wilson DM, Justice C, Thomas R, Sheps S, Macadam M, Brown M. End-of-life care volunteers: a systematic review of the literature. Health services management research. 2005;18(4):244-57.

28. Gillis A, MacLellan M. Service Learning with Vulnerable Populations: Review of the Literature. International Journal of Nursing Education Scholarship. 2010;7(1).

29. Canedo-García A, García-Sánchez J-N, Pacheco-Sanz D-I. A Systematic Review of the Effectiveness of Intergenerational Programs. Frontiers in psychology. 2017;8:1882.

30. Price ML, Surr CA, Gough B, Ashley L. Experiences and support needs of informal caregivers of people with multimorbidity: a scoping literature review. Psychology and Health. 2020;35(1):36-69.

31. Haldane V, Chuah FLH, Srivastava A, Singh SR, Koh GCH, Seng CK, et al. Community participation in health services development, implementation, and evaluation: A systematic review of empowerment, health, community, and process outcomes. PLoS One. 2019;14(5).

32. Ohly H, Gentry S, Wigglesworth R, Bethel A, Lovell R, Garside R. A systematic review of the health and well-being impacts of school gardening: synthesis of quantitative and qualitative evidence. BMC Public Health. 2016;16.

33. Atkinson J-A, Vallely A, Fitzgerald L, Whittaker M, Tanner M. The architecture and effect of participation: a systematic review of community participation for communicable disease control and elimination. Implications for malaria elimination. Malaria Journal. 2011;10:1.

34. Bartone PT, Bartone JV, Violanti JM, Gileno ZM. Peer support services for bereaved survivors: a systematic review. OMEGA-Journal of Death and Dying. 2019;80(1):137-66.

35. Hallett C, Klug G, Lauber C, Priebe S. Volunteering in the care of people with severe mental illness: a systematic review. BMC Psychiatry. 2012;12:-226.

36. Hatton RA, Crane J, Rogers SN, Patterson J. Head and neck cancer peer-to-peer support and quality of life: systematic scoping review. British journal of nursing (Mark Allen Publishing). 2022;31(5):S30-S6.

37. Lu LC, Lan SH, Hsieh YP, Lan SJ. Effectiveness of intergenerational participation on residents with dementia: A systematic review and meta‐analysis. Nursing Open. 2022;9(2):920-31.

38. Smith R, Greenwood N. The impact of volunteer mentoring schemes on carers of people with dementia and volunteer mentors: a systematic review. American Journal of Alzheimer's Disease & Other Dementias®. 2014;29(1):8-17.

39. Casiday R, Kinsman E, Fisher C, Bambra C. Volunteering and health: what impact does it really have. London: Volunteering England. 2008;9(3):1-13.

40. Linning M, Jackson G. Volunteering, Health and Wellbeing. 2018.

41. Patrick R, Henderson‐Wilson C, Ebden M. Exploring the co‐benefits of environmental volunteering for human and planetary health promotion. Health Promotion Journal of Australia. 2022;33(1):57-67.

42. Coleman H, DClinPsy, BSc, Walshe C, PhD, MSc, BNurs , RGN. What are the Emotional Experiences of Being a Volunteer in Palliative and End-of-Life Care Settings? A Systematic Review and Thematic Synthesis. Journal of Pain and Symptom Management. 2021;62(3).

43. Pérez-Ordás R, Nuviala, A., Grao-Cruces, A., & Fernández-Martínez, A. Implementing Service-Learning Programs in Physical Education; Teacher Education as Teaching and Learning Models for All the Agents Involved: A Systematic Review. International Journal of Environmental Research and Public Health. 2021;18(2):669.

44. Sallnow L. Conceptualisation of volunteering in palliative care: a narrative synthesis of the literature. BMJ Supportive & Palliative Care. 2012;2.

45. O’Flynn A, Murphy J, Barrett E. The watersports inclusion games-what are the benefits for volunteers? European Psychiatry. 2021;64(S1):S462-S.

46. Villani M, Kovess-Masféty V. [Peer support programs in mental health in France: Status report and challenges]. L'Encephale. 2018;44(5):457-64.

47. Richards S, Jenkinson C, Dickens A, Jones K, Thompson-Coon J, Taylor R, et al. OP74 Is 'volunteering' a Public Health Intervention: A Systematic Review and Meta-Analysis. Journal of Epidemiology and Community Health. 2013;67.
